# Diabetes Mellitus Increases the Risk of Hepatocellular Carcinoma After Direct-Acting Antiviral Therapy: Systematic Review and Meta-Analysis

**DOI:** 10.3389/fmed.2021.744512

**Published:** 2021-10-18

**Authors:** Szilárd Váncsa, Dávid Németh, Péter Hegyi, Zsolt Szakács, Ádám Farkas, Szabolcs Kiss, Péter Jenő Hegyi, Anna Kanjo, Patrícia Sarlós, Bálint Erőss, Gabriella Pár

**Affiliations:** ^1^Institute for Translational Medicine, Medical School, University of Pécs, Pécs, Hungary; ^2^János Szentágothai Research Centre, University of Pécs, Pécs, Hungary; ^3^Centre for Translational Medicine, Semmelweis University, Budapest, Hungary; ^4^Doctoral School of Clinical Medicine, University of Szeged, Szeged, Hungary; ^5^Heim Pál National Pediatric Institute, Budapest, Hungary; ^6^Department of Gastroenterology, First Department of Medicine, Medical School, University of Pécs, Pécs, Hungary

**Keywords:** chronic hepatitis, glucose metabolism, prognosis, carcinogenesis, liver cancer

## Abstract

**Background:** Hepatitis C virus (HCV)-infected patients treated with direct-acting antivirals (DAAs) are still at risk of developing hepatocellular carcinoma (HCC) after sustained virologic response (SVR). This study aimed to investigate the role of diabetes mellitus (DM) as a potential predictive risk factor in developing *de novo* HCC in HCV-infected patients after DAA treatment.

**Methods:** This study was registered on PROSPERO under registration number CRD42021230457. We performed a systematic search in four medical databases from inception through November 3rd, 2020. Studies were eligible if they reported on HCV-infected patients treated with DAAs and compared the frequency of *de novo* HCC in patients with and without DM. We calculated pooled odds ratios, unadjusted (UHR), and adjusted hazard ratios (AHR) with 95% confidence intervals (CIs) in meta-analysis.

**Results:** We included 30 articles in our systematic review and meta-analysis. DM proved to be a significant risk factor of HCC in DAA-treated HCV patients in unadjusted (*UHR* = 1.44, CI: 1.15–1.79) and adjusted analyses (*AHR* = 1.31, *CI*: 1.06–1.62). In the group of patients achieving SVR after DAA therapy, DM increased the risk of HCC in unadjusted (*UHR* = 1.3, CI: 1.09–1.51) analysis; however, in adjusted results, the risk was non-significant (*AHR* = 1.07, CI: 0.89–1.28). In patients with advanced liver fibrosis, DM was a risk factor for HCC in adjusted (*AHR* = 1.36, CI: 1.03–1.8), but not in unadjusted analysis (*UHR* = 1.11, CI: 0.8–1.42).

**Conclusions:** DM is an independent risk factor of *de novo* HCC after DAA treatment in HCV-infected patients.

**Systematic Review Registration:** https://www.crd.york.ac.uk/prospero/display_record.php?RecordID=230457, identifier: CRD42021230457.

## Introduction

Hepatocellular carcinoma (HCC) is a major cause of cancer-related death worldwide (1). One of the most important causes of HCC is hepatitis C virus (HCV) infection, accounting for 20% of all HCC cases (2). Among patients with chronic HCV, the lifetime prevalence of HCC ranges between 1 and 3%. However, in patients with HCV-related cirrhosis, the proportion of HCC can reach as high as 7% during the follow-up (3).

Since introducing new direct-acting antivirals (DAA), the cure rate among HCV patients approaches 90–100% (4). This high sustained virological response rate (SVR) has been associated with a reduced risk of liver-related and overall mortality. However, according to recent publications, the risk of HCC in DAA-treated patients decreases, but it is not eliminated (4, 5). Therefore, it is clinically important to identify patients carrying a high risk of HCC development after DAA treatment.

The most highlighted risk factor for HCC development is advanced fibrosis or cirrhosis (5). The eradication of HCV may stop the progression of liver disease or even cause fibrosis regression; however, the risk of HCC persists after eradication. This risk is multifactorial, and it can be influenced by other conditions such as chronic alcohol consumption, non-alcoholic fatty liver disease, or type 2 diabetes mellitus (T2DM) (6).

Previously T2DM was associated with a higher risk of HCC, regardless of other toxic factors, such as alcohol consumption or steatosis (7). In HCV patients, this increased risk was present both in pre- and post-interferon treatment (8). However, in DAA-treated HCV patients, the role of T2DM as a risk factor of HCC development is still contradictory. The study of Benhammou et al. (9) found that diabetes was independently associated with increased risk of mortality [hazard ratio (HR) = 1.25, 95% confidence interval (CI): 1.16–1.48] and HCC (*HR* = 1.32, 95% CI: 1.01–1.72) in DAA-treated HCV patients, however, in a population not excluding previous HCC cases. Another large retrospective study, multivariate analysis found an increased risk of HCC in patients with DM (*HR* = 2.52, 95% CI: 1.06–5.87) (10). Other studies did not find an association between pre-DAA treatment DM and risk of HCC in sustained virological responders (11, 12). To our knowledge, no meta-analysis assessed the connection between DM and HCC development after DAA therapy for HCV.

Our study aimed to assess the risk of HCC in patients with DM after DAA therapy for HCV to resolve the contradictory results in this topic. We hypothesized that DM increases the risk of HCC development after DAA treatment.

## Methods and Materials

We conducted our systematic review and meta-analysis according to the recommendations proposed by the Cochrane Collaboration (13), and we report our study following the Preferred Reporting Items for Systematic Reviews and Meta-Analyses (PRISMA) 2020 Statement (Supplementary Table 1) (14). The study protocol was registered onto the International Prospective Register of Systematic Reviews (PROSPERO, registration number CRD42021230457, see https://www.crd.york.ac.uk/prospero). We did not deviate from the initial protocol.

### Systematic Search

MEDLINE (via PubMed), Embase, Cochrane Central Register of Controlled Trials (CENTRAL), and Web of Science databases were searched for relevant publications. We searched the mentioned databases from inception to November 3rd, 2020. The search strategy included the following keywords: {[“direct acting antiviral” OR boceprevir OR glecaprevir OR grazoprevir OR paritaprevir OR simeprevir OR telaprevir OR voxilaprevir OR daclatasvir OR elbasvir OR ledipasvir OR ombitasvir OR pibrentasvir OR velpatasvir OR dasabuvir OR sofosbuvir] AND “hepatitis C” AND (carcinoma OR cancer OR tumor OR malign OR neoplasm)}. No language or any other restrictions were used during the search.

### Selection and Eligibility of Studies

The yield of the search was imported into a reference management program, EndNote v9.0 (Clarivate Analytics, Philadelphia, PA, USA). After removing duplicate studies, two independent authors searched the library by title, abstract, and full-text for relevant articles. Disagreements were resolved by discussion at the level of abstract and full-text selection, while at the level of title selection, the selected studies were merged.

Eligible full-text articles reported on HCV patients (P) treated with any DAA treatment and compared the outcome of patients with and without DM (E and C). Regarding the definition of DM, we used the one reported in each included article. Since most of the articles did not define the type of DM, we did not differentiate between the types. The outcome of interest (O) was the incidence of *de novo* HCC. Studies with a mean follow-up period of at least six months were included in our analysis because of the time necessary for HCC development. All included articles excluded previous cases of HCC. Previous unsuccessful treatment with IFN-based therapy was not an exclusion criterion; however, studies were excluded if patients received combined DAA and IFN treatment. Eligible articles reported on the proportion of HCC in patients with and without DM or reported their results using unadjusted or adjusted Cox hazard models. Regarding study design, descriptive studies were excluded. In the case of overlapping populations, we selected the studies with the greatest number of participants.

### Data Extraction

Two review authors extracted the data independently. Disagreements were resolved by consensus. A standardized form was used for data extraction, which included: first author, the year of publications, study population, study period, study site (country), study design, demographic characteristics of the included patients, follow-up period, characteristics of hepatitis C virus infection, relative measure for the risk of HCC in patients with and without DM, event rate in patients with and without DM, and information for assessing the risk of bias in the studies. Unadjusted and adjusted results were extracted separately.

### Data Synthesis

All statistical analysis of the data was conducted using the Stata 15.1 SE program package (Stata Corp LLC, College Station, TX, USA) and Comprehensive Meta-Analysis (version 3, Biostat Inc., Englewood, NJ, USA). We calculated pooled hazard ratios (HRs) with 95% confidence intervals (CIs) from unadjusted and adjusted results (UHR and AHR, respectively), and pooled odds ratios (ORs) with 95% CIs from 2 x 2 tables (HCC vs. no-HCC, and DM vs. no-DM groups). UHR and AHR were pooled separately. Random effects model was used to calculate the pooled estimates using the DerSimonian-Laird method (15). If the HR was reported with an asymmetrical CI, the article was excluded from the meta-analysis.

Heterogeneity was tested with I^2^ and χ^2^ tests; *p*-value < 0.1 indicated statistically significant heterogeneity. Heterogeneity are presented in the results section and on the forest plots. Publication bias was assessed by the Egger's test and by visual analysis of Funnel plots. To investigate heterogeneity, we performed a random-effect meta-regression analysis between the mean follow-up period after DAA treatment and the rate of HCC in each included article. Publication bias assessment and meta-regression were performed if there were at least 10 studies included in the analysis. Except for heterogeneity, a *p*-value < 0.05 was considered statistically significant.

A subgroup analysis was carried out with articles reporting only on patients achieving a SVR after DAA treatment (Occurrence of hepatocellular carcinoma in patients with sustained virological response). Furthermore, articles reporting on patients with advanced liver fibrosis with and without SVR were analyzed in a subgroup (Occurrence of hepatocellular carcinoma in patients with advanced liver fibrosis (METAVIR F3-F4) and Occurrence of hepatocellular carcinoma in patients with advanced liver fibrosis (METAVIR F3-F4) achieving sustained virological response, respectively). In sub-group analysis advanced liver fibrosis was defined as METAVIR stage F3 and/or F4 (16), since articles reported on the stage of fibrosis differently.

### Risk of Bias Assessment in Individual Studies

Two review authors independently assessed the risk of bias for each included study using the Quality in Prognostic Studies (QUIPS) tool (17). Disagreements were resolved by consensus. Methodological details of the assessment are summarized in Supplementary Appendix 1. We used the Risk-of-bias VISualization (robvis) web-based tool to visualize summary plots of the assessed domains (18).

## Results

### Search and Selection

**Figure 1:**
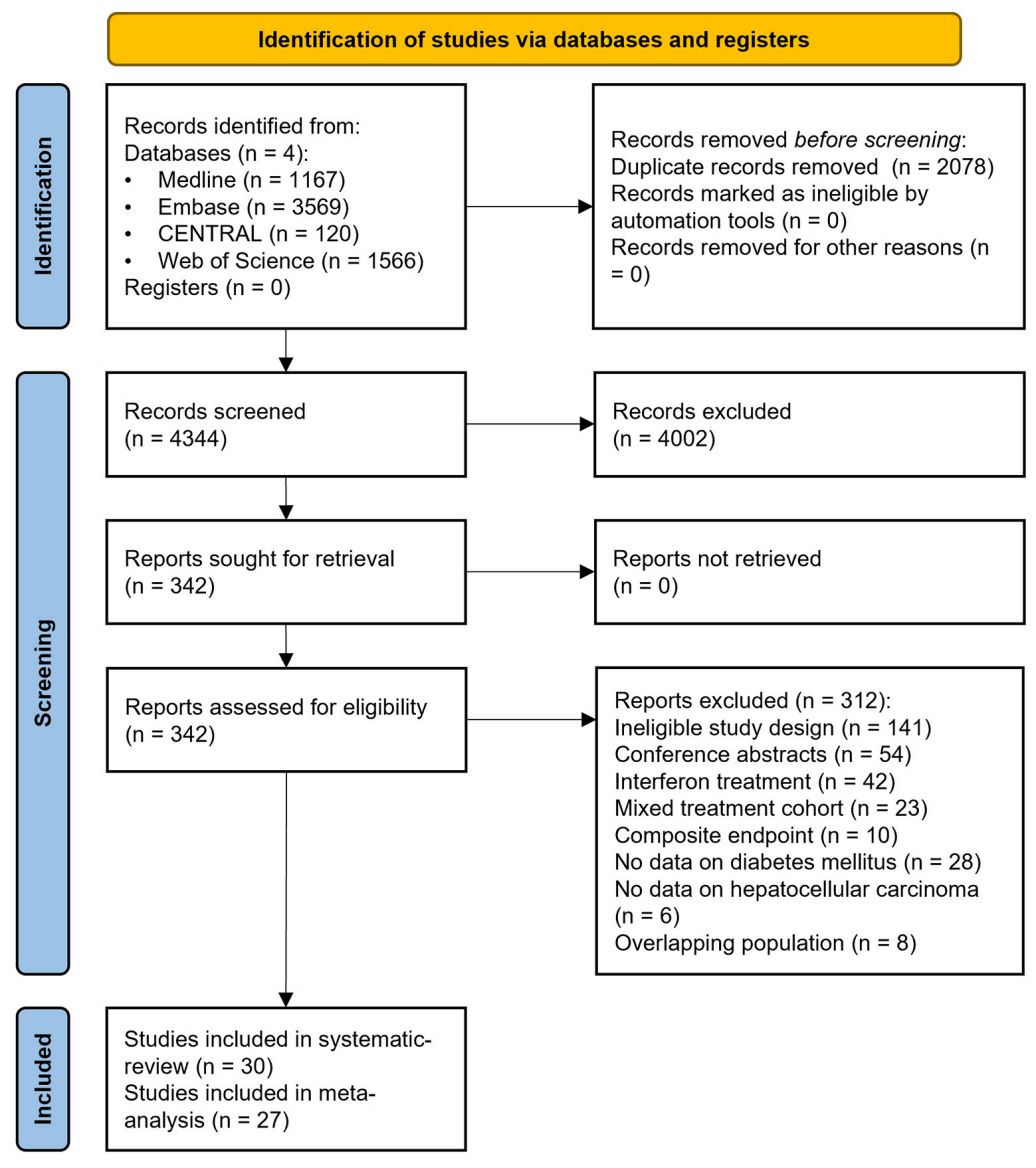
Preferred Reporting in Systematic Reviews and Meta-analyses 2020 (PRISMA) flowchart showing the selection process.

We detailed the selection process in Figure 1. Our search strategy yielded a total of 6,422 records. After duplicate removal and selection by title and abstract, 342 articles were eligible for full-text assessment. We included 30 observational studies in our qualitative synthesis (10, 12, 19–43); however, we excluded three articles from the quantitative synthesis due to asymmetric CIs (11, 44, 45).

### Basic Characteristics of the Included Studies

The main characteristics of the included articles are summarized in Table 1. The eligibility criteria of each included article are summarized in Supplementary Table 2. Out of the 30 included articles, 17 were published from Europe, nine from Asia, two from North America, one from South America, and one from Africa. The mean follow-up period ranged between 6 and 45 months, and the rate of HCC ranged between 1 and 34.3%. Generally, *de novo* HCC was assessed every 3 to 6 months after DAA treatment using abdominal imaging (ultrasound, computed tomography, or magnetic resonance). The follow-up policies of the included articles are summarized in Supplementary Table 3.

**Table 1:** Basic characteristics of included studies.

**References**	**Study site**	**Study type**	**No of patients (female %)**	**Age (years)^†^**	**Follow up period (months)^†^**	**F3/F4 rate (subgroup)^‡^**	**Genotype (GT)**	**DM (% of total)**	**SVR rate**	**Overall HCC rate**
Calvaruso et al. (19)	Italy	prospective	2249 (43)	65.4	14.0	100%	GT1a/1b/2/3/4/other	30	95.2%	3.5%
Ciancio et al. (20)	Italy	prospective	893 (42)	59.6	44.5	67%	GT1a/1b/2a/2c/3/4/5/6	16	100%	2.5%
Conti et al. (21)	Italy	prospective	344 (40)	63.0	6.0	100%	GT1/2/3/4	17	91.6%	3.2%
Degasperi et al. (10)	Italy	retrospective	505 (40)	63.0	25.0	100%	GT1b/other	19	96.4%	5.5%
Faillaci et al. (22)	Italy	prospective	155 (33)	62.2	N/A	100%	GT1a/1b/2/3/4	18	90.3%	13.6%
Gardini et al. (23)	Italy	retrospective	416 (42)	63.3	18.2	100%	GT1a/1b/2/3/4	24	<100%	7%
Ide et al. (24)	Japan	prospective	2552 (61)	64.6	22.6	30%	GT1/2	20	100%	2.8%
Janjua et al. (25)	Canada	retrospective	3905 (33)	N/A	12.0	14% (0%, 100%)	GT1/2/3/other	20	92.5%	1%
Kanwal et al. (26)	USA	retrospective	18076 (4)	61.6	35.0	38% (0%, 100%)	GT1/2/3/other	43	100%	3%
Lleo et al. (27)	Italy	prospective	1766 (38)	61.7	12.0	100%	GT1a/1b/2/3/4	20	95.1%	2.8%
Alonso Lopez et al. (28)	Spain	prospective	993 (45)	61.7	45.0	100%	N/A	17	100%	3.6%
Mariño et al. (29)	Spain	retrospective	1123 (40)	59.3	19.6	100%	GT1/other	19	95.2%	6.4%
Mecci et al. (30)	UK	prospective	245 (25)	57.0	32.4	100%	GT1/3/other	29	80.4%	34.3%
Mettke et al. (31)	Germany	prospective	158 (45)	59.0	14.7	100%	GT1a/1b/2/3/4/other	23	100%	3.8%
Nagata et al. (44)	Japan	prospective	752 (55)	69.0	21.6	33%	GT1a/1b/2a/2b/3a/other	15	96.0%	1.1%
Nakagawa et al. (32)	Japan	prospective	947 (54)	65.9	24.2	28%	N/A	12	100%	2.7%
Ogasawara et al. (33)	Japan	retrospective	398 (61)	70.0	39.6	51%	GT1b	10	100%	4.8%
Ogawa et al. (11)	Japan	prospective	1675 (56)	66.0	17.0	18%	GT1/2	18	100%	2.7%
Ozeki et al. (34)	Japan	retrospective	769 (59)	64.0	35.0	19%	GT1/2	12	100%	2.3%
Piñero et al. (12)	Latin America	prospective	1400 (52)	58.0	16.0	56% (100%)	GT1a/1b/2/3/4/other	15	97.0%	2.1%
Pons et al. (35)	Spain	prospective	572 (51)	63.7	34.8	100%	GT1/2/3/4	22	100%	4.4%
Quaranta et al. (36)	Italy	prospective	3114 (44)	59.0	38.9	N/A	GT1/2/3/4/5	14	94.9	1.4%
Rinaldi et al. (37)	Italy	prospective	985 (45)	67.0	12.0	100%	GT1/2/3/4	13	98.1%	3.6%
Romano et al. (38)	Italy	prospective	3917 (38)	58.1	17.9	100%	GT1a/1b/2/3/4/other	11	93.9%	1.4%
Sangiovanni et al. (39)	Italy	prospective	1161 (41)	65.0	17.0	100%	GT1a/1b/2/3/4	21	96%	4.1%
Shiha et al. (40)	Egypt	prospective	2372 (48)	N/A	23.6	100%	GT4	20	100%	4.6%
Tani et al. (45)	Japan	retrospective	1088 (50)	68.0	13.8	18%	GT1a/1b/2/3/4/6	16	100%	2.6%
Tayyab et al. (41)	Pakistan	prospective	662 (51)	50.0	12.0	49%	GT3/other	28	91.9%	6.3%
Watanabe et al. (42)	Japan	prospective	1174 (54)	66.0	24.0	N/A	GT1/2	15	100%	2.8%
Yoshimasu et al. (43)	Japan	retrospective	211 (48)	63.0	21.0	N/A	GT1a/1b/2a/2b	13	91.5%	1%

† *mean or median*.

‡ *advanced liver fibrosis and cirrhosis based on METAVIR scores (F3 and F4)*.

*HCC, hepatocellular carcinoma; GT, genotype; SVR, sustained viral response*.

### Occurrence of Hepatocellular Carcinoma in all the Included Articles

Overall, 13 articles reported unadjusted and eight adjusted HRs on the risk of HCC in patients with and without DM. The odds of HCC at the end of the follow-up period were reported in 19 articles.

**Figure 2:**
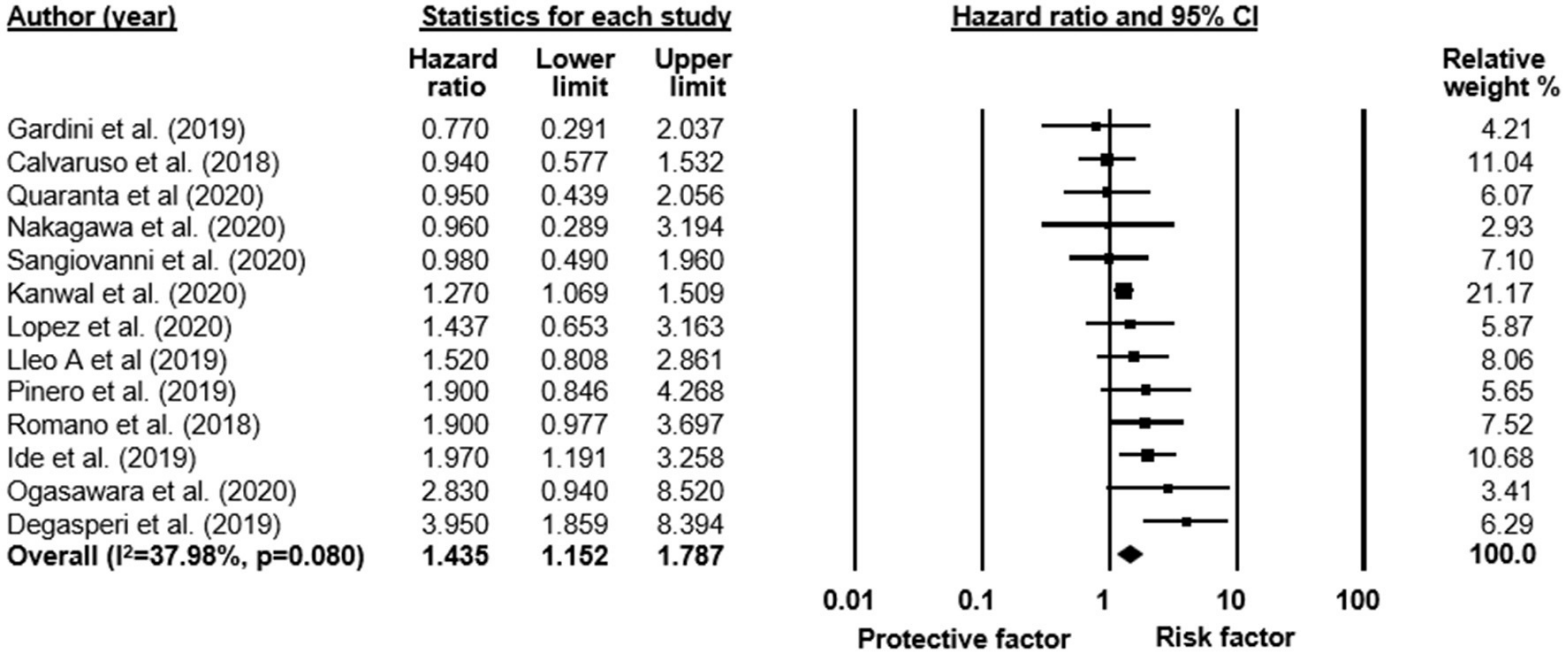
Forest plot with pooled unadjusted hazard ratio, representing the risk of HCC in all patients with and without DM after HCV treatment with DAA therapy.

Based on 13 articles with 35,373 patients, our results show that DM is associated with an increased risk of HCC after DAA treatment in unadjusted results (*UHR* = 1.44, CI: 1.15–1.79; heterogeneity *I*^2^ = 38%, *p* = 0.08; Figure 2). A similar result was found in the pool of eight studies (*n* = 30,416) reporting adjusted analysis (*AHR* = 1.31, CI: 1.06–1.62; *I*^2^ = 18.7%, *p* = 0.282; Supplementary Figure 1). The odds to develop HCC were also higher at the end of the follow-up period in patients with DM than those without DM (5.2 vs. 3%; respectively, *OR* = 1.68, CI: 1.35–2.08; *I*^2^ = 30%, *p* = 0.106; Supplementary Figure 2).

Meta-regression analysis showed no significant correlation between mean follow-up months and the risk of HCC in patients with DM (Supplementary Figures 3, 4).

### Occurrence of Hepatocellular Carcinoma in Patients With Sustained Virological Response

**Figure 3:**
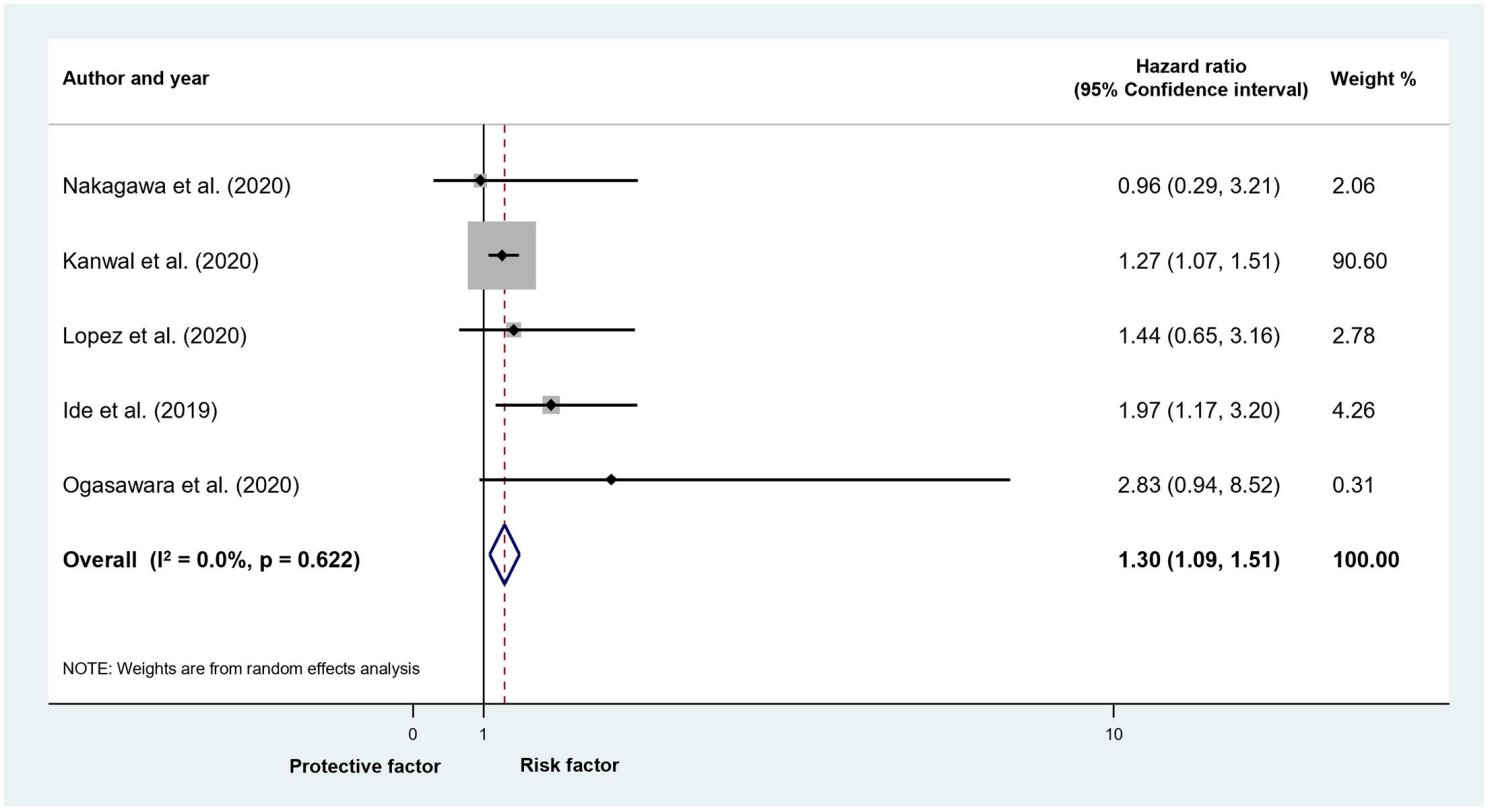
Forest plot with pooled unadjusted hazard ratio, representing the risk of HCC in patients with and without DM who achieved sustained virological response after HCV treatment with DAA therapy.

In the subgroup analysis of five studies reporting on patients who achieved SVR after DAA treatment (*n* = 22,791 patients), the risk of HCC was higher in patients with DM (*UHR* = 1.3, CI: 1.09–1.51; *I*^2^ = 2.9%, *p* = 0.404; Figure 3). The difference was not significant in adjusted models, although only three articles (*n* = 21,229) were included in this analysis (*AHR* = 1.07, CI: 0.89–1.28; *I*^2^ = 0%, *p* = 0.890; Supplementary Figure 5). We found a significant difference in the proportion of HCC in patients with DM compared to those without DM after successful DAA treatment (4.9 vs. 3%; *OR* = 1.71, CI: 1.22–2.4; *I*^2^ = 28.8%, *p* = 0.198; Supplementary Figure 6).

### Occurrence of Hepatocellular Carcinoma in Patients With Advanced Liver Fibrosis (METAVIR F3-F4)

In the subgroup of patients with advanced liver fibrosis, eight articles with 12,373 reported on unadjusted and six articles on adjusted results (*n* = 30,705). DM was associated with an increased risk of HCC in adjusted (*AHR* = 1.36, CI: 1.03–1.8; *I*^2^ = 34.4%, *p* = 0.178; Supplementary Figure 7), but not in unadjusted analyses (*UHR* = 1.11, CI: 0.8–1.42; *I*^2^ = 0%, *p* = 0.505; Supplementary Figure 8). The odds of HCC were higher in DM patients with advanced liver fibrosis compared to patients without DM (5.5 vs. 4.3%; *OR* = 1.51, CI: 1.15–1.99; *I*^2^ = 39.1%, *p* = 0.073; Supplementary Figure 9).

### Occurrence of Hepatocellular Carcinoma in Patients With Advanced Liver Fibrosis (METAVIR F3-F4) Achieving Sustained Virological Response

We were unable to assess the risk in unadjusted and adjusted hazard models. The rate of HCC was not significantly higher in patients with DM compared to those without DM (5.7 vs. 3.9%; *OR* = 1.67, CI: 0.91–3.07; *I*^2^ = 51%, *p* = 0.106; Supplementary Figure 10).

### Risk of Bias Assessment

The results of the risk of bias assessment of individual studies are summarized in Supplementary Table 4. Out of the six assessed domains, the prognostic factor measurement was attributed moderate or high risk of bias in 24 of the included articles due to missing definition of DM (Supplementary Figures 11, 12). In Supplementary Table 5 we summarized the parameters included in multivariate adjustment in each included article.

The assessment of publication bias could only be performed in the case of three comparisons. We did not detect the presence of publication in these comparisons (see Supplementary Figures 13–15 for funnel plots and Egger's test alfa value).

## Discussion

In our meta-analysis, we aimed to analyze the association between DM and the risk of HCC after all DAA treatments for HCV infection, as successful antiviral therapy is known to reduce but does not eliminate the risk of developing HCC. We found an increased risk of HCC among patients with DM in unadjusted and adjusted analyses. In the subgroups with SVR or advanced liver fibrosis, the risk was also increased.

Based on current guidelines, a thorough clinical assessment is recommended every six months after successful DAA treatment in patients with advanced fibrosis or cirrhosis (METAVIR F3 and F4). The European Association for the Study of the Liver (EASL), in their 2020 recommendations on treatment of hepatitis C, highlights DM as a co-factor for liver disease and recommends a closer follow-up of these patients after treatment for HCV (4). However, the guideline does not refer to an increased risk of HCC in patients with DM. The American Association for the Study of Liver Diseases (AASLD) recommends surveillance for HCC only in patients with cirrhosis. For non-cirrhotic patients, the recommended follow-up is as in non-HCV infected patients, without specification on DM (46).

Our results are consistent with previous results. In the general population, T2DM was associated with a moderately increased risk of HCC incidence (risk ratio = 2.23, 95% CI: 1.68–2.96) (47). We found a similar, 1.7-fold increased odds of HCC after DAA treatment in patients with DM, which was comparable in sub-groups as well. On the other hand, diabetes with HCV infection increases the risk of HCC by 2 to 3-folds, which is higher than the risk in patients with DM after DAA treatment (48). In a review of prospective studies, the risk of HCC after DAA treatment was between 2.1 and 5.4% after median follow-up periods of few months and 33.4 months (49). In our results, HCC occurrence was similar in patients with DM between 4.9 and 5.7%, while in patients without DM being lower, between 3 and 4.3%.

The risk of HCC after DAA treatment is most probably multifactorial. In an experimental study, authors found a decreased expression of inhibitory checkpoint receptors upon innate immune cells after DAA therapy in HCV patients (50). This may favor decreased immune surveillance against tumor cells. HCV infection-induced epigenetic modifications associated with HCC risk, such as H3K27ac, persist after DAA treatment (51). On the other hand, liver comorbidities such as metabolic associated fatty liver disease or alcohol consumption were highlighted as risk factors of HCC (5). Metabolic risk factors, such as metabolic syndrome, obesity, or insulin resistance, may further increase the risk of HCC through the presence of low-grade chronic inflammation (48). Furthermore, T2DM contributes to fibrosis progression after DAA therapy, which is a risk of HCC (52).

There is a two-way association between HCV infection and DM (53). Hepatitis C infection was showed to significantly increase the odds of DM, compared to different control groups (54). It is estimated that up to 33% of chronic hepatitis C patients have DM, which is influenced by increased age, male gender, duration of infection, and other risk factors (55). The underlying mechanisms include hepatic steatosis, an increase in reactive oxygen agents, and inflammatory cytokines, which lead to insulin resistance (53). On the other hand, DM leads to the progression of hepatic fibrosis in HCV infection (56), which starts early in infection and involves oxidative stress, inflammation, and oncogenesis (57).

The risk of HCC was different in the included articles. In the study of Benhammou et al. (9), an increased risk of *de novo* and recurrent HCC was found in T2DM patients without cirrhosis (*AHR* = 1.32, 95%CI: 1.01–1.72) but not in those with cirrhosis (*AHR* = 1.06, 95%CI: 0.92–1.23). However, patients with previous HCC were not excluded. Overall, in this study, the authors did not find an increased risk of HCC in patients with T2DM, but they concluded that pre-DAA diabetes increases mortality and liver-related events in patients with and without SVR. Two other studies analyzed the subgroup of patients without cirrhosis (25, 26), neither of them found a significant difference between patients with and without T2DM (*AHR* = 3.08, 95%CI: 0.93–10.17; *AHR* = 0.98, 95%CI: 0.67–1.44, respectively). We did not have enough data to analyze the risk in patients without cirrhosis.

The highest risk of HCC among patients with DM was found in the study of Degasperi et al. (10); they reported a 3-year cumulative incidence of 16% in patients with diabetes and 4% in patients without diabetes (*p* < 0.001). The proportion of HCC increased with other risk factors; in diabetic male patients with a liver stiffness >30 kPa, 50% developed *de novo* HCC. The longest follow-up in the included studies was a median of 45 months (28). The risk of HCC did not differ in this study in patients with DM. However, based on our analysis, the mean follow-up period did not correlate with the risk of HCC.

One study analyzed the combined risk of *de novo* and recurrent HCC in diabetic patients by multivariate Cox regression analysis (20). In this study, the duration of DM > 10 years, family history of DM, and no improvement of DM were not associated with an increased incident HCC (*p* > 0.05), while insulin therapy was an unfavorable predictor (*HR* = 4.11, 95% CI: 1.20–14.13). Iuliano et al. (58) reported on the risk of HCC based on the duration of DM. In patients with and without metabolic syndrome, the risk increased with longer duration; however, the risk was higher in those with metabolic syndrome (*p* = 0.002). On the other hand, Mecci et al. (30) demonstrated that both in the early (<6 months) and late (>6 months) HCC groups, DM was present in a higher proportion compared to the non-HCC group (36 and 33% vs. 19%, respectively); although the difference was significant only in the late group (*p* < 0.05). These results suggest that the increased risk in patients with DM is constantly present.

Lastly, Romano et al. (38) analyzed the risk of HCC in the presence of one or more risk factors. In patients with DM, the cumulative proportion of HCC was 3%, while DM with hepatitis B surface antigen (HBsAg) positivity and APRI score ≥2.5 increased the risk up to 18%. In addition to these, Child-Turcotte Pugh B further increased the proportion of HCC to 29%. Based on these results, the presence of DM with additional risk factors will result in a much higher increase in the proportion of HCC.

The proper control of DM should be carried out in both pre- and post-DAA treatment to decrease the risk of liver fibrosis progression and HCC development. Successful treatment of HCV with DAA contributes to a reduced glycated hemoglobin level (mean difference = 0.45, 95% CI: 0.3–0.6), which may further decrease the risk of HCC (59). Furthermore, in a systematic review of metformin's protective effect in diabetic patients, authors found a reduced risk of HCC (*OR* = 0.47; 95% CI: 0.28–0.8) (60).

### Strengths and Limitation

Our study has several strengths. First, we registered the pre-study protocol and followed it wholly. Second, we managed to include a substantial number of articles with a high number of patients. Third, we could pool adjusted HR results. On the other hand, our study has limitations.

Most importantly, the definition of DM was missing in most of the articles, carrying a high risk of bias. Studies mainly included patients with advanced liver disease or a high proportion of them; therefore, it is hard to generalize our results. The follow-up period was relatively short in most of the studies and was different among studies; it is possible that the higher risk of HCC would drop after longer follow-ups. Authors used other methods to screen for HCC; however, all of them used abdominal imaging. Besides prospective studies, we included retrospective cohort analyses as well. Lastly, some of the results carry a moderate risk of statistical heterogeneity.

### Implication for Practice

Overall, successful antiviral treatment reduces but does not eliminate the risk of HCC. Additionally, the risk of HCC is higher in patients with advanced liver fibrosis and DM, so they should be followed up more closely after HCV eradication with DAAs. Other conditions potentially increasing liver fibrosis progression must be assessed and handled correctly.

### Implication for Research

Further studies are needed to clarify the risk of HCC in DAA-treated DM populations, based on the duration, treatment, and complications of DM. It is a question of whether the adequate treatment of DM decreases the risk of HCC in patients with SVR. Lastly, cost-effectiveness studies should be initiated to determine the proper follow-up period (3 vs. 6 months) in a high-risk group of patients.

## Conclusion

There is an increased risk of HCC development in patients with DM compared to patients without DM after DAA treatment for HCV infection.

## Data Availability Statement

The original contributions presented in the study are included in the article/Supplementary Material, further inquiries can be directed to the corresponding author.

## Author Contributions

SV: conceptualization, project administration, formal analysis, writing—original draft. DN: conceptualization, formal analysis, visualization, writing—original draft. PH: conceptualization, funding acquisition, writing—review and editing. ZS and ÁF: conceptualization, data curation, writing—review and editing. SK: conceptualization, methodology, writing—review and editing. PH, AK, and PS: conceptualization, writing—review and editing. BE: conceptualization, methodology, visualization, writing—original draft. GP: conceptualization, supervision, writing—original draft. All authors certify that they have participated sufficiently to take public responsibility for the content, including participation in the concept, design, analysis, writing, or revision of the manuscript.

## Funding

Funding was provided by an Economic Development and Innovation Operative Programme Grant (GINOP-2.3.4-15-2020-00010) and by a Human Resources Development Operational Programme Grant (EFOP-3.6.2-16-2017-00006, EFOP-3.6.1.-16-2016-00004), both co-financed by the European Union (European Regional Development Fund) within the framework of the Széchenyi 2020 Program. Furthermore, funding was provided by the ÚNKP-20-3-I-PTE-604, a New National Excellence Program of the Ministry for Innovation and Technology from the source of the National Research, Development, and Innovation Fund (for SV). Sponsors had no role in the design, data collection, analysis, interpretation, and manuscript preparation.

## Conflict of Interest

The authors declare that the research was conducted in the absence of any commercial or financial relationships that could be construed as a potential conflict of interest.

## Publisher's Note

All claims expressed in this article are solely those of the authors and do not necessarily represent those of their affiliated organizations, or those of the publisher, the editors and the reviewers. Any product that may be evaluated in this article, or claim that may be made by its manufacturer, is not guaranteed or endorsed by the publisher.
