# Supplementary material for: Diabetes Mellitus Increases the Risk of Hepatocellular Carcinoma After Direct-Acting Antiviral Therapy: Systematic Review and Meta-Analysis
Source: Front Med (Lausanne). 2021 Oct 18;8:744512. doi: 10.3389/fmed.2021.744512 (PMC8558240; doi:10.3389/fmed.2021.744512)
Supplement: Supplementary file 1 [file Data_Sheet_1.docx]

Supplementary Material

Diabetes mellitus increases the risk of hepatocellular carcinoma after direct-acting antiviral therapy: systematic review and meta-analysis

Szilárd Váncsa^1,2^, Dávid Németh^1^, Péter Hegyi^1,2,3^, Zsolt Szakács^1^, Ádám Farkas^1^, Szabolcs Kiss^1,4^, Péter Jenő Hegyi^1^, Anna Kanjo^1,4,5^, Patrícia Sarlós^6^, Bálint Erőss^1,3^, Gabriella Pár^6*^

^1^ Institute for Translational Medicine, Medical School, University of Pécs, Pécs, Hungary;

^2^ János Szentágothai Research Centre, University of Pécs, Pécs, Hungary;

^3^ Centre for Translational Medicine, Semmelweis University, Budapest, Hungary;

^4^ Doctoral School of Clinical Medicine, University of Szeged, Szeged, Hungary;

^5^ Heim Pál National Pediatric Institute, Budapest, Hungary;

^6^ Division of Gastroenterology, First Department of Medicine, Medical School, University of Pécs, Pécs, Hungary;

# TABLE OF CONTENT

**Supplementary Appendix 1.**

**Supplementary Figure 1-10.** Supplementary forest plots and meta-regression

**Supplementary Figure 11-12.** Summary plots for risk of bias assessment

**Supplementary Figure 13-15.** Assessment of publication bias

**Supplementary Table 1.** PRISMA checklist

**Supplementary Table 2.** Eligibility criteria in each included article

**Supplementary Table 3.** HCC surveillance strategy and definition of diabetes mellites in each included article

**Supplementary Table 4.** Risk of bias assessment using the QUIPS tools

**Supplementary Table 5.** Parameters included in multivariate adjustment in each included article

**References**

# Supplementary Appendix 1.

Overall ratings for each domain were assigned as carrying ‘low’ (green), ‘moderate’ (yellow) or ‘high’ (red) risk of bias, based on the items included in each domain.

Study participation measurement: (1) low risk of bias was attributed if authors adequately described the source population, including methods to identify patients and eligibility criteria. The description of the time period and place of recruitment were also added to low risk of bias; (2) moderate risk of bias was attributed if a part of the above listed descriptions were missing; (3) high risk of bias was attributed if baseline characteristics, eligibility criteria, time and place of recruitment were not described.

Study attrition assessment was performed in the case of prospective studies: (1) low risk of bias was attributed if the proportion of baseline sample was available, also if the reason for lost to follow-up was detailed; (2) moderate risk of bias was attributed if a part of the above listed criteria were missing; (3) high risk of bias was attributed if data was missing for the above mentioned criteria. We did not assess attrition bias in retrospective studies (N/A – not attributable).

Prognostic factor measurement: (1) low risk of bias was attributed, if the definition of diabetes mellitus was based on clear criteria (glycated hemoglobin level, antidiabetic treatment, followed guideline, etc.); (2) moderate risk of bias was attributed if data on diabetes was acquired from anamnesis; (3) high risk of bias was attributed if there was no definition for diabetes mellitus.

Outcome measurement: (1) low risk of bias was attributed if the patients were followed-up regularly after direct acting antiviral treatment, and hepatocellular carcinoma was investigated using abdominal imaging modalities; (2) moderate risk of bias was attributed if a part of the above mentioned criteria were missing; (3) high risk of bias was attributed if the follow-up policy was not described or it was inadequate. Only studies excluding patients with HCC prior to DAA treatment were included in our analysis.

Study confounding measurement: (1) low risk of bias was attributed if important potential confounders were described and accounted for in the analysis (i.e., appropriate adjustment); (2) moderate risk of bias was attributed if some of the important confounders were not measured; (3) high risk of bias was attributed if studies did not provide data on confounding factors.

Statistical analysis measurement was assessed separately for hazard ratio and odds ratios: (1) for hazard ratio, low risk of bias was attributed if Cox hazard model was used, and the used methodology was clearly described; (2) moderate risk of bias was attributed if the statistical analysis was not clearly described; (3) high risk of bias was attributed if the used methodology was not described. Odds ratios were calculated from raw data reported in all eligible studies, therefore we attributed low risk of bias for this domain.

# Supplementary Figures

**
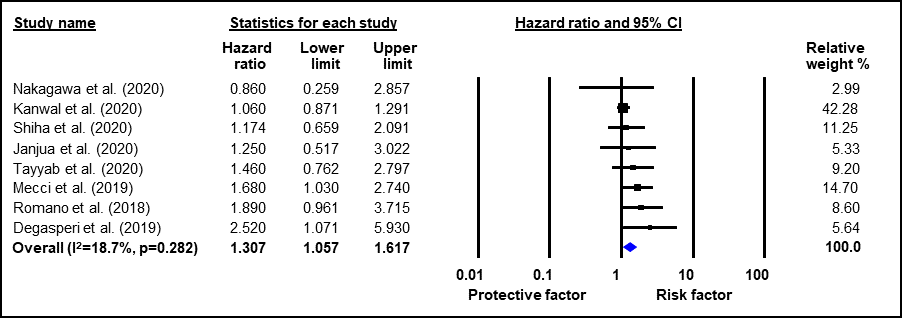
Supplementary Figure 1.** Forest plot with pooled adjusted hazard ratio, representing the risk of hepatocellular carcinoma in patients with or without diabetes mellitus after hepatitis C treatment with direct acting antiviral therapy (heterogeneity I^2^=18.65%, p=0.282).

**Supplementary Figure 2.** Forest plot with pooled odds ratio (OR), representing the risk of hepatocellular carcinoma in patients with or without diabetes mellitus (DM) after hepatitis C treatment with direct acting antiviral therapy.

Protective factor Risk factor

**Supplementary Figure 3.** Meta-regression analysis between mean follow-up period and risk of hepatocellular carcinoma in patients with diabetes mellitus in each included article (p=0.714)

Hazard ratio

Odds ratio

**Supplementary Figure 4.** Meta-regression analysis between mean follow-up period and odds of hepatocellular carcinoma in patients with diabetes mellitus in each included article (p=0.308)

**
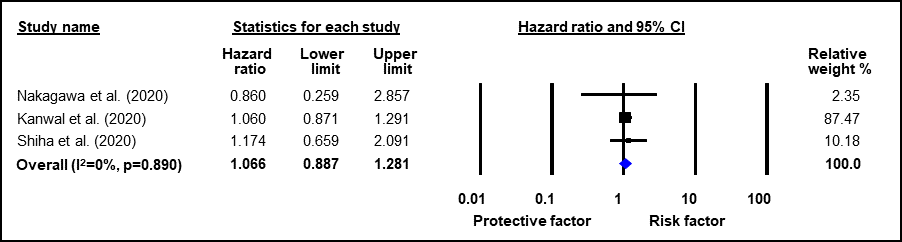
Supplementary Figure 5.** Forest plot with pooled adjusted hazard ratio, representing the risk of hepatocellular carcinoma in patients with or without diabetes mellitus who achieved sustained virological response after hepatitis C treatment with direct acting antiviral therapy (heterogeneity I^2^=0.0%, p=0.890)

**Supplementary Figure 6.** Forest plot with pooled odds ratio (OR), representing the risk of hepatocellular carcinoma in patients with or without diabetes mellitus (DM) who achieved sustained virological response after hepatitis C treatment with direct acting antiviral therapy.

Protective factor Risk factor

**
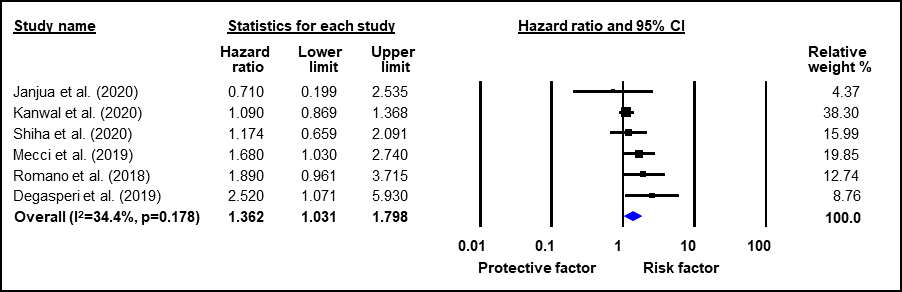
Supplementary Figure 7.** Forest plot with pooled adjusted hazard ratio, representing the risk of hepatocellular carcinoma in patients with advanced liver fibrosis with or without diabetes mellitus after hepatitis C treatment with direct acting antiviral therapy (heterogeneity I^2^=34.38%, p=0.178).

**Supplementary Figure 8.** Forest plot with pooled unadjusted hazard ratio, representing the risk of HCC in patients with and without DM, and with advanced liver fibrosis (METAVIR F3 or F4) after HCV treatment with DAA therapy

HR (95% CI)

Protective factor Risk factor

**Supplementary Figure 9.** Forest plot with pooled odds ratio (OR), representing the risk of hepatocellular carcinoma in patients with advanced liver fibrosis with or without diabetes mellitus (DM) after hepatitis C treatment with direct acting antiviral therapy.

Protective factor Risk factor

**Supplementary Figure 10.** Forest plot with pooled odds ratio (OR), representing the risk of hepatocellular carcinoma in patients with advanced liver fibrosis with or without diabetes mellitus (DM) who achieved sustained virological response after hepatitis C treatment with direct acting antiviral therapy.

Protective factor Risk factor

**
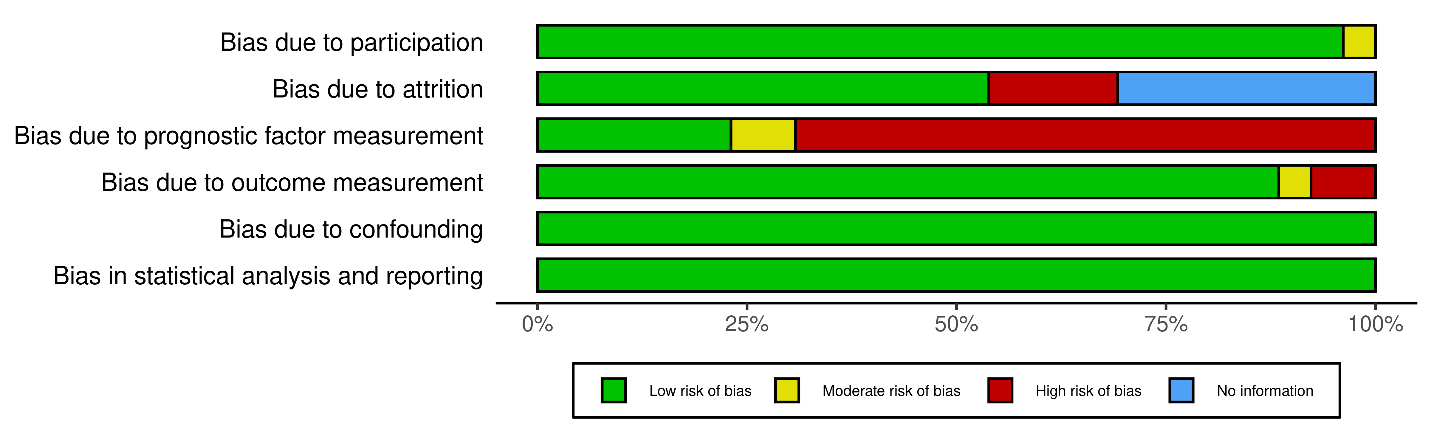
Supplementary Figure 11.** Summary plot for risk of bias assessment for studies reporting on hazard ratios of HCC occurrence

**
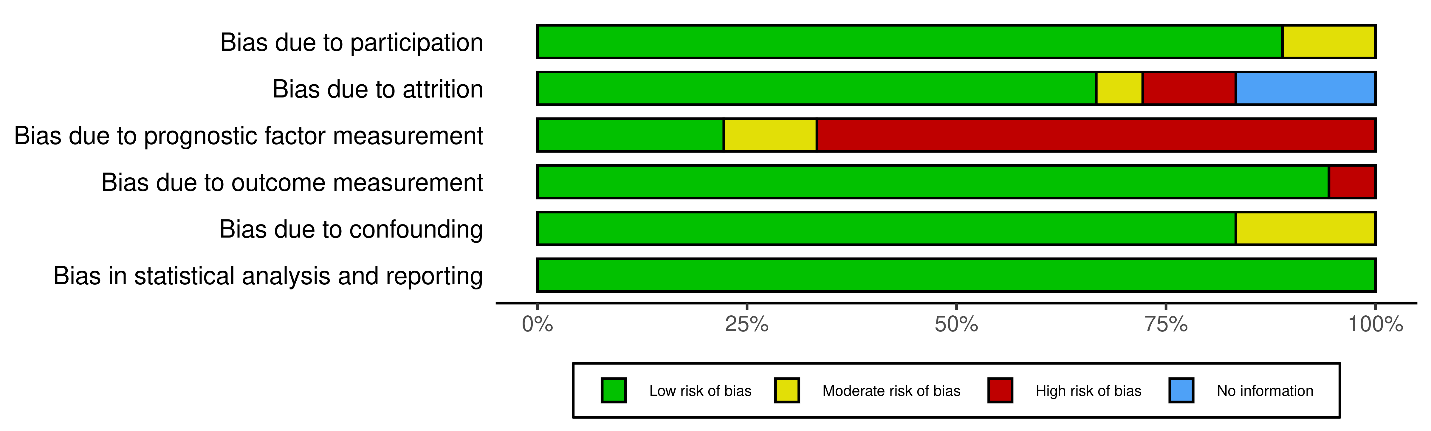
Supplementary Figure 12.** Summary plot for risk of bias assessment for studies reporting on the odds of HCC occurrence

**Supplementary Figure 13.** Funnel plot for Figure 1 (Egger’s test alfa = 0.415)

**Supplementary Figure 14.** Funnel plot for Supplementary Figure 2 (Egger’s test alfa = 0.942)

**Supplementary Figure 15.** Funnel plot for Supplementary Figure 9 (Egger’s test alfa = 0.583)

# Supplementary Tables

**Supplementary Table 1.** Preferred Reporting Items for Systematic Reviews and Meta-Analyses (PRISMA) 2020 checklist (1)

| **Section and Topic** | **Item #** | **Checklist item** | **Location where item is reported** |
| --- | --- | --- | --- |
| **TITLE** | | |  |
| Title | 1 | Identify the report as a systematic review. | Page 1 |
| **ABSTRACT** | | |  |
| Abstract | 2 | See the PRISMA 2020 for Abstracts checklist. | Pages 1-2 |
| **INTRODUCTION** | | |  |
| Rationale | 3 | Describe the rationale for the review in the context of existing knowledge. | Page 2 |
| Objectives | 4 | Provide an explicit statement of the objective(s) or question(s) the review addresses. | Page 2 |
| **METHODS** | | |  |
| Eligibility criteria | 5 | Specify the inclusion and exclusion criteria for the review and how studies were grouped for the syntheses. | Page 3 |
| Information sources | 6 | Specify all databases, registers, websites, organisations, reference lists and other sources searched or consulted to identify studies. Specify the date when each source was last searched or consulted. | Page 3 |
| Search strategy | 7 | Present the full search strategies for all databases, registers and websites, including any filters and limits used. | Page 3 |
| Selection process | 8 | Specify the methods used to decide whether a study met the inclusion criteria of the review, including how many reviewers screened each record and each report retrieved, whether they worked independently, and if applicable, details of automation tools used in the process. | Page 3 |
| Data collection process | 9 | Specify the methods used to collect data from reports, including how many reviewers collected data from each report, whether they worked independently, any processes for obtaining or confirming data from study investigators, and if applicable, details of automation tools used in the process. | Page 3 |
| Data items | 10a | List and define all outcomes for which data were sought. Specify whether all results that were compatible with each outcome domain in each study were sought (e.g. for all measures, time points, analyses), and if not, the methods used to decide which results to collect. | Page 3 |
|  | 10b | List and define all other variables for which data were sought (e.g. participant and intervention characteristics, funding sources). Describe any assumptions made about any missing or unclear information. | Page 3 |
| Study risk of bias assessment | 11 | Specify the methods used to assess risk of bias in the included studies, including details of the tool(s) used, how many reviewers assessed each study and whether they worked independently, and if applicable, details of automation tools used in the process. | Page 4 |
| Effect measures | 12 | Specify for each outcome the effect measure(s) (e.g. risk ratio, mean difference) used in the synthesis or presentation of results. | Pages 3-4 |
| Synthesis methods | 13a | Describe the processes used to decide which studies were eligible for each synthesis (e.g. tabulating the study intervention characteristics and comparing against the planned groups for each synthesis (item #5)). | Pages 3-4 |
|  | 13b | Describe any methods required to prepare the data for presentation or synthesis, such as handling of missing summary statistics, or data conversions. | N/A |
|  | 13c | Describe any methods used to tabulate or visually display results of individual studies and syntheses. | Pages 3-4 |
|  | 13d | Describe any methods used to synthesize results and provide a rationale for the choice(s). If meta-analysis was performed, describe the model(s), method(s) to identify the presence and extent of statistical heterogeneity, and software package(s) used. | Pages 3-4 |
|  | 13e | Describe any methods used to explore possible causes of heterogeneity among study results (e.g. subgroup analysis, meta-regression). | Pages 3-4 |
|  | 13f | Describe any sensitivity analyses conducted to assess robustness of the synthesized results. | N/A |
| Reporting bias assessment | 14 | Describe any methods used to assess risk of bias due to missing results in a synthesis (arising from reporting biases). | Page 4 |
| Certainty assessment | 15 | Describe any methods used to assess certainty (or confidence) in the body of evidence for an outcome. | N/A |
| **RESULTS** | | |  |
| Study selection | 16a | Describe the results of the search and selection process, from the number of records identified in the search to the number of studies included in the review, ideally using a flow diagram. | Page 4 |
|  | 16b | Cite studies that might appear to meet the inclusion criteria, but which were excluded, and explain why they were excluded. | Page 4 |
| Study characteristics | 17 | Cite each included study and present its characteristics. | Page 4, Table 1 |
| Risk of bias in studies | 18 | Present assessments of risk of bias for each included study. | Page 5 |
| Results of individual studies | 19 | For all outcomes, present, for each study: (a) summary statistics for each group (where appropriate) and (b) an effect estimate and its precision (e.g. confidence/credible interval), ideally using structured tables or plots. | Pages 4-5 |
| Results of syntheses | 20a | For each synthesis, briefly summarise the characteristics and risk of bias among contributing studies. | Pages 4-5 |
|  | 20b | Present results of all statistical syntheses conducted. If meta-analysis was done, present for each the summary estimate and its precision (e.g. confidence/credible interval) and measures of statistical heterogeneity. If comparing groups, describe the direction of the effect. | Pages 4-5 |
|  | 20c | Present results of all investigations of possible causes of heterogeneity among study results. | Pages 4-5 |
|  | 20d | Present results of all sensitivity analyses conducted to assess the robustness of the synthesized results. | Pages 4-5 |
| Reporting biases | 21 | Present assessments of risk of bias due to missing results (arising from reporting biases) for each synthesis assessed. | N/A |
| Certainty of evidence | 22 | Present assessments of certainty (or confidence) in the body of evidence for each outcome assessed. | N/A |
| **DISCUSSION** | | |  |
| Discussion | 23a | Provide a general interpretation of the results in the context of other evidence. | Pages 5-7 |
|  | 23b | Discuss any limitations of the evidence included in the review. | Page 7 |
|  | 23c | Discuss any limitations of the review processes used. | Page 7 |
|  | 23d | Discuss implications of the results for practice, policy, and future research. | Pages 7-8 |
| **OTHER INFORMATION** | | |  |
| Registration and protocol | 24a | Provide registration information for the review, including register name and registration number, or state that the review was not registered. | Page 2  CRD42021230457 |
|  | 24b | Indicate where the review protocol can be accessed, or state that a protocol was not prepared. | PROSPERO |
|  | 24c | Describe and explain any amendments to information provided at registration or in the protocol. | N/A |
| Support | 25 | Describe sources of financial or non-financial support for the review, and the role of the funders or sponsors in the review. | Page 8 |
| Competing interests | 26 | Declare any competing interests of review authors. |  |
| Availability of data, code and other materials | 27 | Report which of the following are publicly available and where they can be found: template data collection forms; data extracted from included studies; data used for all analyses; analytic code; any other materials used in the review. |  |

**Supplementary Table 2.** Eligibility criteria in each included article

| **Author (year)** | **Inclusion criteria (“verbatim”)** | **Exclusion criteria (“verbatim”)** |
| --- | --- | --- |
| Calvaruso et al. 2018 (2) | Patients with cirrhosis treated with DAAs who were consecutively included in the RESIST-HCV database from March 1, 2015 to November 30, 2016 and were available for SVR assessment 12 weeks after therapy at the time of analysis (February 28, 2017). | Patients with previous diagnosis of HCC, previous liver transplantation (LT), or on an LT waiting list were excluded. Patients who withdrew from therapy, those not evaluable for SVR, and those who had not undergone at least one ultrasound after the start of DAA therapy were not assessed for outcomes. |
| Ciancio et al. 2020 (3) | age >18 years; positive HCV-RNA by polymerase chain reaction (PCR); chronic liver disease assessed by transient elastography (TE) and aspartate aminotransferase to platelet ratio (APRI) score or liver biopsy | Lack of written informed consent, patients on waiting list for orthotopic liver transplant (OLT), post-OLT patients, presence of hepatocellular carcinoma (HCC) not referred to or not responding to curative treatment confirmed by the modified Response Evaluation Criteria in Solid Tumours (mRECIST), presence of ascites, concomitant liver diseases such as haemochromatosis, Wilson's disease, drug-related liver disease, autoimmune hepatitis, HBsAg carriership, Human immunodeficiency Virus (HIV) infection, primary biliary cholangitis and alpha-1-antitrypsin deficiency. |
| Conti F et al. 2016 (4) | Patients with Child-Pugh class A or B liver cirrhosis, without history of previous HCC or with history of complete response to surgical resection or loco-regional ablation of previous HCC, and patients with a METAVIR F3 fibrosis score, assessed by liver histology or transient elastography result >10 kPa. | Since our analysis was restricted to patients with liver cirrhosis, 82 patients were excluded because of a METAVIR score of less than F3 or a transient elastography result of less than 12 kPa. |
| Degasperi et al. 2019 (5) | Consecutive HCV patients with cirrhosis starting DAA treatment between December 2014 and 2016. | Child-Pugh-Turcotte (CPT) C score, whose treatment was not reimbursed by Italian National Health System outside the liver transplant waiting list; human immunodeficiency virus coinfection; and active HCC at baseline (DAA start), whose treatment was not allowed by Italian prescription rules, patients with baseline atypical or uncharacterized (nodule size <1 cm or diagnostic algorithm not concluded) liver nodules according to 2012 HCC EASL recommendations. |
| Faillaci et al. 2018 (6) | Patients had Child-Pugh class A or B LC; no history of previous HCC or histories of complete response for at least 3 months after surgical resection or locoregional ablation of previous HCC, and/or an F3 METAVIR fibrosis score, determined by liver histology or transient elastography result between 10 and 12.5 kilopascals (kPa). | N/A |
| Gardini et al. 2019 (7) | All the consecutive HCV-infected cirrhotic patients treated with DAAs. | Patients with previous liver transplantation or with treated HCC without radiological complete response before starting DAAs were excluded. |
| Ide et al. 2019 (8) | Chronic hepatitis C patients with or without cirrhosis who were treated with DAAs and achieved a SVR at 12 weeks (SVR12). | HCC prior to DAA treatment, hepatitis B virus surface antigen, or other forms of liver diseases. |
| Janjua et al. 2020 (9) | This analysis included patients who filled at least one prescription of HCV treatment. | All diagnoses of HCC before the index date and those occurring within 6 months of the index date were excluded. |
| Kanwal et al. 2020 (10) | Patients 18 years or older who achieved SVR with DAA treatment. | Patients who failed to achieve SVR, patients with missing FIB-4 or APRI measurements at the baseline or landmark time. |
| Lleo et al. 2019 (11) | Consecutively treated with DAA, Diagnosis of cirrhosis was established by the presence of at least one of the following elements: previous liver biopsy with stage 4 fibrosis by METAVIR score (or equivalent Ishak), esophageal and/or gastric varices at endoscopy, liver stiffness measurement (LSM) higher than 12.5 kPa at Transient Elastometry (FibroScan®). Functional class of cirrhosis was attributed by Child–Turcotte–Pugh (CTP) and MELD score. | Patients with Metavir F3 and Ishak 3–4, HBV, or HIV co-infection, and/or active HCC were excluded. Further, we excluded from the study patients with significant alcohol intake. Importantly patients with active HCC were not allowed to receive DAA treatment according to AIFA rules; therefore, all patient included in the study had a rigorous definition of complete radiologic response (CRR) before treatment. Further, patients on waiting list for Liver Transplant were excluded from the study. |
| Lopez et al. 2020 (12) | The study included compensated patients with HCV under HCC screening after achieving SVR as recommended in clinical guidelines who had one or more of the following criteria: (1) patients with advanced fibrosis defined by a liver stiffness measurement (LSM) by transient elastography (TE) (FibroScan; Echosens SA, Paris, France) >9.5 kPa(22); (2) patients with unequivocal ultrasound signs of cirrhosis (blunted nodular liver surface and/or splenomegaly) in combination with platelets count lower than 150 × 109/L; and (3) patients with histologic diagnosis of F3 or F4 fibrosis. | Patients who were coinfected with human immunodeficiency virus and hepatitis B virus and also those with past or current history of liver decompensation or with a previous diagnosis of HCC. |
| Marino et al. 2019 (13) | Inclusion criteria were: 1) liver cirrhosis; 2) abdominal imaging assessment before antiviral therapy to discard HCC; 3) treatment with an all-oral DAA combination. Cirrhosis was defined according to any of the following criteria: liver stiffness measurement (LSM)≥14Kpa within the last 12 months; liver biopsy; clinical evidence of portal hypertension (esophageal varices and/or liver decompensation) or at least two ultrasonographical criteria of cirrhosis (liver surface nodularity, enlarged spleen or portal vein diameter>12mm). | Exclusion criteria were: 1) patients not meeting the cirrhosis diagnostic criteria; 2) prior HCC; 3) previous liver transplantation; 4) patients receiving IFN as part of the antiviral regimen; 5) Antiviral therapy initiated outside the study centers. All data were double-checked by expert hepatologists (ZM, MR, SL) to confirm the accuracy of follow-up results and inclusion/exclusion criteria. |
| Mecci et al. 2019 (14) | Entry to the English early access programme specified that all patients had to have either a diagnosis of hepatic decompensation in the past or have current evidence of CTP score B or C. The HCV Research UK database was interrogated for all cases of de novo HCC diagnosed from the start of the early access programme until 15 June 2017 regardless of diagnostic modality. A control group (two controls per case) of early access programme patients with no subsequent diagnosis of HCC was then selected based on frequency matching for age, gender, Child‐Turcotte‐Pugh score and length of follow‐u. | Patients with a prior liver transplant or HCC diagnosis before the onset of DAA therapy were excluded. |
| Mettke et al. 2017 (15) | This study included patients with chronic hepatitis C and liver cirrhosis which were recruited from the regular liver outpatient clinic of Hannover Medical School in Germany. | Exclusion criteria were: (1) lack of cirrhosis; (2) history of liver transplanation or HCC; (3) liver imaging not available/accessible at start of therapy and/or at end of observation period; (4) observation period<12 weeks; (5) interferon-based treatment during the 6 months before start of observation. |
| Nagata et al. 2017 (16) | Patients over 18 years, infected with chronic hepatitis C virus (HCV) infection, detectable plasma HCV RNA levels and were treated previously. | Patients were excluded if they were positive for serum hepatitis B surface antigen, co-infection with human immunodeficiency virus, or other causes of hepatocellular injury (such as alcoholic liver injury, autoimmune hepatitis, primary biliary cholangitis, or a history of treatment with hepatotoxic drugs). |
| Nakagawa et al. 2020 (17) | Inclusion criteria: patients over 20 years old, with chronic HCV infection and with detectable plasma HCV RNA levels, and who were treated. | Patients who were positive for serum hepatitis B surface antigen, co-infected with human immunodeficiency virus, or with other causes of hepatocellular injury (such as alcoholic liver injury, autoimmune hepatitis, primary biliary cholangitis, or a history of treatment with hepatotoxic drugs) were excluded. |
| Ogasawara et al. 2020 (18) | (i) the patient tested positive for anti-HCV by a third-generation enzyme immunoassay (Chiron Corp, Emeryville, CA) andHCVRNAby quantitative analysis before antiviral therapy; (ii) the patient had SVR24 (i.e. negative results for HCVRNA at 24 weeks after the cessation of antiviral therapy) by the Cobas TaqMan HCV test (Roche Diagnostics, Tokyo, Japan); (iii) the patient was confirmed to be free of HCC before and during antiviral therapy; (iv) the patient had no history ofEV rupture; (v) the patient was negative for decompensated liver cirrhosis; (vi) the patient was infected with a single genotype of HCV genotype 1b, as tested by the HCV Monitor genotype assay (BML, Tokyo, Japan); (vii) the patient was negative for hepatitis B surface antigen, as confirmed by a chemiluminescent enzyme immunoassay (Abbott Laboratories, Tokyo, Japan); (viii) the patient was free of coinfection with the human immunodeficiency virus; (ix) the patient had a history ofmild-to-moderate alcohol intake (estimated lifetime cumulative alcohol intake of 500 kg); (x) the patient was free ofother types of hepatitis; and (xi) the patient was confirmed to have no hemochromatosis, Wilson disease, primary biliary cholangitis, alcoholic liver disease, or autoimmune liver disease; (xii) the drug regimen were: (a) 24 weeks of dual therapy with 60 mg daclatasvir (an NS5A inhibitor) once daily combined with 100 mg asunaprevir (an NS3/4A protease inhibitor) twice daily, (b) 12 weeks of dual therapy with 90 mg ledipasvir (an NS5A inhibitor) once daily combined with 400 mg sofosbuvir (a nucleotide NS5B polymerase inhibitor) once daily, and (c) 12 weeks of a triple-therapy regimen of 25 mg ombitasvir (an NS5A inhibitor) once daily, 150mg paritaprevir (an NS3/4Aprotease inhibitor) once daily, and 100 mg ritonavir once daily. The selection was based on the following criteria: (xiii) the patient underwent LS measurement at least twice before and the end of the treatment (EOT) during the follow-up period; (xiv) the follow-up periodwas >0.5 year after the achievement ofSVR; and (xv) no HCC occurrence was found within 0.5 year after the achievement ofSVR. | Patients who were lost to follow up were excluded. |
| Ogawa et al. 2018 (19) | N/A | (1) under age 20 at the initiation of treatment, (2) decompensated cirrhosis (Child‐Pugh B or C), (3) concomitant human immunodeficiency virus or hepatitis B virus infection, (4) excessive active alcohol consumption and (5) history of organ transplantation. |
| Ozeki et al. 2020 (20) | N/A | Patients with decompensated cirrhosis, chronic kidney disease (CKD) stage ≥4, concomitant human immunodeficiency virus or hepatitis B virus (HBV) infection, comorbid liver disease associated with autoimmunity, excessive alcohol consumption (daily ethanol consumption was ≥60 g/day), history of HCC, or HCC detected during the DAA treatment and within 24 weeks after EOT were excluded. |
| Pinero et al. 2019 (21) | Consecutive adult patients (>18 years of age) with chronic HCV infection. Patients with any degree of liver fibrosis treated with all‐oral DAA were eligible. All patients who received at least one pill of DAAs were included in the study as part of an intention to treat analysis. | Patients with prior solid organ transplantation or previous diagnosis of HCC were excluded. |
| Pons et al. 2020 (22) | Suspected cACLD defined by LSM ≥10 kPa and no prior decompensation (ascites, variceal bleeding, hepatic encephalopathy or jaundice) according to Baveno VI definition;8 ii) confirmed SVR 12 weeks after finishing therapy; and iii) Child-Pugh class A. | Exclusion criteria: i) LSM was not available before starting therapy; ii) they had history of prior HCC or developed HCC before confirming SVR; iii) they had prior liver transplant or iv) they had concomitant coinfection with HBV and/or HIV. |
| Quaranta et al. 2020 (23) | DAA treated patients with pre-treatment diagnosis of liver cirrhosis who achieved SVR12. HIV/HCV coinfected patients and HCV monoinfected patients with known HIV negative status were also included. | Patients with a history of decompensated cirrhosis or liver transplantation prior to treatment were excluded by this analysis as reported in the methods section. |
| Rinaldi et al. 2019 (24) | Inclusion criteria were HCV-RNA serum positivity and fbrosis stage≥F3 according to Metavir score (the Italian reimbursement criteria were applicable only for patients with F3–F4 fbrosis), assessed either by liver biopsy or transient elastography (TE). Te TE was performed by Fibroscan® (Echosens, Paris, France), according to standard criteria. | N/A |
| Romano et al. 2018 (25) | Baseline fibrosis stage ≥F3 according to METAVIR classification29 as assessed by transient elastography or liver biopsy. | Child-Turcotte-Pugh (CTP)-C, liver transplantation before DAAs, history or presence of HCC, follow-up <4 weeks after starting DAAs. |
| Sangiovanni et al. 2020 (26) | Patients with no history of HCC (group 1) and patients with a history of HCC and a radiological complete response to cancer therapy (group 2). | Child-Pugh (CP) C class outside the liver transplant waiting list and active HCC. |
| Shiha et al. 2020 (27) | Patients 18 years or older with HCV who received DAA, have advanced liver fibrosis (F3) or cirrhosis (F4), and have no history or current HCC. | Patient with either HBV or HIV co‐infection, or with a history of previous IFN‐treatment, liver transplantation, renal impairment, liver cell failure and other malignancies were excluded. |
| Tani et al. 2020 (28) | Achieved sustained virologic response and who had no history of HCC treatment. | i) Patients co-infected with hepatitis B virus or human immunodeficiency virus, or patients with other liver diseases, including primary biliary cholangitis and autoimmune hepatitis; ii) patients with decompensated cirrhosis, since IFN-free DAA treatment was not approved for these patients in Japan; and iii) patients with a previous history of HCC or patients who did not achieve SVR12 following DAA treatment. |
| Tayyab et al. 2020 (29) | All consecutive persons who provided informed consent, were eligible for the study and free of HCC were enrolled. | N/A |
| Watanabe et al. 2019 (30) | N/A | Non-SVR, history of HCC before beginning antiviral therapy were excluded from this study. |
| Yoshimasu et al. 2019 (31) | N/A | 1) patients with treated HCC without a complete radiologic response; 2) patients receiving IFN as part of their treatment regimen; and 3) patients who had not undergone a 6-month observation period after DAA treatment. |

**Supplementary Table 3.** HCC surveillance strategy and definition of diabetes mellites in each included article

| **Author (year)** | **HCC surveillance (“verbatim”)** | **Diabetes mellitus definition** |
| --- | --- | --- |
| Calvaruso et al. 2018 (2) | When a focal lesion was identified in the liver by US, HCC was confirmed by imaging (computed tomography and/or magnetic resonance imaging) and/or biopsy examination, as suggested by guidelines. | N/A |
| Ciancio et al. 2020 (3) | Patients developing any suspicious liver lesion revealed by liver US were further examined with a complementary imaging method to confirm or exclude HCC. | Criteria recommended by the Expert Committee on the Diagnosis and Classification of Diabetes Mellitus. |
| Conti F et al. 2016 (4) | At the end of antiviral therapy, 12 and 24 weeks thereafter, patients repeated abdominal ultrasound evaluation. | N/A |
| Degasperi et al. 2019 (5) | HCC surveillance was performed by US scan every 6 months in patients with no previous HCC history or with a complete response (CR) to a previous HCC treatment >2 years; patients with a CR 2 years performed a computed tomography every 3 months. CR was defined as 2 consecutive negative imaging assessments following HCC treatment (computed tomography scan performed 1 and 3 months after HCC treatment according to mRECIST criteria). In patients under US-based HCC surveillance, baseline US scan was performed within 3 months before DAA start. As a consequence, all patients included in the study had at least 1 radiologic examination performed in the 3 months preceding DAA start, because absence of HCC or CR to a previous HCC treatment was required by Italian prescription rules to receive antiviral therapy. According to the previously mentioned exclusion criteria, all patients with baseline imaging showing a focal lesion (either atypical or uncharacterized nodule) were excluded from the study. HCC was diagnosed, staged, and treated according to international recommendations. | N/A |
| Faillaci et al. 2018 (6) | Before starting antiviral therapy and on a 6-month basis, all patients underwent hepatic ultrasound (US) examination. In cases of suspected liver lesions, computed tomography or magnetic resonance imaging was performed. All patients with HCC underwent US-guided liver biopsy of the tumor and surrounding nontumoral liver tissue at the time of diagnosis. For patients with previously treated HCC, samples from US-guided liver biopsy of the primary HCC and surrounding cirrhotic tissue were available, because they had been collected in a prospective study of HCC aggressiveness (Clinicaltrials.gov: NCT01657695). In case of recurrence, patients underwent repeat US-guided liver biopsy. | N/A |
| Gardini et al. 2019 (7) | All patients with no HCC history underwent abdomen ultrasound (US). If a potential focal lesion was detected in the liver, the diagnostic work-up was completed with contrast-enhanced ultrasonography (CEUS), and a subsequent computerized tomography (CT) scan or magnetic resonance imaging (MRI) was performed to exclude the presence of HCC. All the patients with a history of HCC underwent ultrasound and CT scan or MRI to exclude recurrent HCC. | N/A |
| Ide et al. 2019 (8) | Among abdominal imaging tests such as ultrasonography (US), contrast computed tomography (CT), and contrast magnetic resonance imaging (MRI), at least US was required as an instrumental test to assess HCC development at the time of patient enrollment. Contrast CT or contrast MRI was not mandatory for patients before DAA therapy, because our primary goal was to obtain “real-world” data from reliable core hospitals reflecting typical clinical practice in hepatology. | Diagnostic criteria established by the Japan Diabetes society. |
| Janjua et al. 2020 (9) | HCC surveillance is performed based on guidelines. HCC diagnosis was based on records in the BC Cancer Registry (BCCR) database using International Classification of Disease–Oncology version 3 (ICD-O-3), topography code C22.0 and histology codes 81703-81753. BCCR diagnoses are based on either histological or radiological evidence of HCC. All diagnoses of HCC before the index date and those occurring within 6 months of the index date were excluded. | Diagnostic codes and/or prescription drug records in administrative health datasets. |
| Kanwal et al. 2020 (10) | HCC was defined based on two or more instances of ICD-9 (155.0) or ICD-10 codes (C22.0, C22.8, C22.9, D01.5) in CDW or any instance of HCC recorded in the CCR. | International Classification of Diseases -9/10 |
| Lleo et al. 2019 (11) | Cirrhotic patients with no prior history of HCC underwent ultrasound (US) screening by a specialized dedicated specialist and alpha-fetoprotein (AFP) determination every 6 months, all patients had a recent assessment before starting DAA (maximum time lapse 3 months). Per protocol, every single patient with previous HCC had CRR assessed by CT Scan or MR 40 days after HCC treatment, repeated after 3 months, and every 6 months after that. Further, CT Scan or MR was repeated before starting DAA (maximum of 3 months). | N/A |
| Lopez et al. 2020 (12) | During follow‐up, any liver event was recorded. HCC was diagnosed according to Barcelona Clinic Liver Cancer guidelines. Patients were followed up until the first liver event, HCC, or death occurred or until November 1st, 2019, when data were finally updated. | N/A |
| Marino et al. 2019 (13) | The presence of liver cancer was ruled out by abdominal imaging assessment in all patients before entering the study. The detection of benign entities or non-characterized nodules together with their size and pattern were registered. Non-characterized nodules were defined as nodules ≤10 mm or nodules >10 mm but in which HCC diagnosis was ruled out before starting DAA by contrast-enhanced ultrasound, computed tomography, magnetic resonance or biopsy. Baseline nodules and those appearing during follow-up were evaluated in each center following their clinical practice and ultimately sent to Hospital Clinic for a central evaluation (AD, EB, CA). The imaging dates, type of images and the description of liver nodules at each time-point of the radiologic evaluation were registered.  Radiologic assessment was divided into baseline (pre-DAA images [pre-DAAi]) and follow-up (post-DAA images [post- DAAi]) images. For those patients who did not develop liver nodules after starting DAA, we registered the first post-DAAi (defined as the first imaging study performed after DAA initiation) and the last post-DAAi (defined as the last radiologic evaluation at the time of the database lock) ruling out the presence of nodules. For those patients who developed liver nodules after starting DAA, the first and successive post-DAAi to assess tumor progression or response were registered. Two pathologists with more than 10 years’ experience in HCC (AD, MS) performed the central pathology evaluation of biopsies. Central radiologists and pathologists were blinded to the patient clinical evolution. | N/A |
| Mecci et al. 2019 (14) | The HCV Research UK database was interrogated for all cases of de novo HCC diagnosed from the start of the early access programme until 15 June 2017 regardless of diagnostic modality. | N/A |
| Mettke et al. 2017 (15) | HCC was diagnosed by two positive images and/or histology. | N/A |
| Nagata et al. 2017 (16) | All patients were followed up every 3 to 12 months. Diagnostic imaging, either by ultrasonography (US), computed tomography (CT), or magnetic resonance imaging (MRI), was performed at intervals of between 3 to 12 months. | N/A |
| Nakagawa et al. 2020 (17) | All patients were followed up every 3–12 months by measurements of blood cell counts and liver biochemistry. The surveillance protocols were in accordance with the standard of care in Japan and an HCC diagnosis was made based on positive results of typical vascular patterns, as revealed by either contrast-enhanced ultrasonography (US), computed tomography (CT), magnetic resonance imaging (MRI) or angiography. | Diabetes mellitus (DM) was defined as recurrent or persistent hyperglycemia, and was diagnosed when the patient demonstrated any one of the following: fasting plasma glucose level 126 mg/dL, plasma glucose levels 200 mg/dL with or without 75-g oral glucose tolerance test (75-g OGTT), glycated hemoglobin (HbA1c) C 6.5%, and the use of DM medication. |
| Ogasawara et al. 2020 (18) | Surveillance for HCC was conducted according to the Practice Guidance of the American Association for the Study of Liver Disease. | N/A |
| Ogawa et al. 2018 (19) | All patients were examined for HCC by abdominal ultrasonography, dynamic computed tomography and/or magnetic resonance imaging at baseline and every 3‐6 month after the initiation of treatment. Serum α‐fetoprotein (AFP) was determined every 3 months for each patient. The surveillance protocols were in accordance with the Japanese standards of care. | N/A |
| Ozeki et al. 2020 (20) | N/A | Patients with a HbA1c value ≥6.5% or those undergoing treatment with antidiabetic drugs or insulin |
| Pinero et al. 2019 (21) | All patients were under strict HCC surveillance in order to exclude HCC diagnosis prior to DAA initiation, according to international guidelines. (AASLD, EASL). | N/A |
| Pons et al. 2020 (22) | Data on abdominal ultrasound performed within 6 months before starting therapy was also collected. Once SVR was confirmed, patients underwent abdominal ultrasound and laboratory work-up every 6 months, as per standard clinical practice, and LSM at 12 months after finishing treatment. | N/A |
| Quaranta et al. 2020 (23) | Clinical outcomes evaluated following the SVR12 included the appearance of incident HCC and the frst occurrence of a decompensating event. | N/A |
| Rinaldi et al. 2019 (24) | The baseline HCC screening for all patients enrolled in our cohort was performed according to the European Association for the Study of Liver (EASL) guidelines. An abdominal ultrasound (US) was performed before starting the antiviral therapy (within 1 month). Each US was performed by an experienced operator. | N/A |
| Romano et al. 2018 (25) | All patients underwent a complete clinical and laboratory evaluation including an abdomen ultrasonography during the three months preceding the initiation of DAA therapy. If a suspected focal lesion was identified in the liver, a full diagnostic work-up was completed with another complementary imaging technique and, when indicated by liver biopsy. | N/A |
| Sangiovanni et al. 2020 (26) | Surveillance for early diagnosis of HCC was performed by abdominal US every 6 months in patients without previous HCC, while surveillance for early detection of HCC recurrence was performed by contrast-enhanced CT or MRI every 3 months for 2 years after achievement of CR and by abdominal US in patients with CR achieved 2 or more years earlier. | N/A |
| Shiha et al. 2020 (27) | In each follow‐up visit, hematological and biochemical parameters were determined, together with abdominal ultrasound. | N/A |
| Tani et al. 2020 (28) | N/A | N/A |
| Tayyab et al. 2020 (29) | All enrolled persons were followed at monthly intervals during the course of treatment and for 24 weeks after completion of treatment, and at three monthly intervals thereafter for at least 12 months after treatment completion. | DM self reported |
| Watanabe et al. 2019 (30) | All DAA-treated patients were followed at 3- to 6-monthintervals, and biochemical and virological markers and blood counts were monitored. Patients also underwent ultrasonography (US) or helical dynamic computed tomography (CT) every 3–6 months to screen for HCC. When new lesions were detected or suspected at the time of US or dynamic CT, patients were further examined by magnetic resonance imaging (MRI) or hepatic angiography. Hepatocellular carcinoma was diagnosed by the presence of typical hypervascular characteristics on angiography, in addition to the ﬁndings of dynamic CT or MRI. If no typical ﬁndings of HCC were observed, the diagnosis was con-ﬁrmed by ﬁne-needle aspiration biopsy followed by histological examination. | HbA1c>6.5% or taking antidiabetes drugs or insulin preparationsbefore starting DAA therapy |
| Yoshimasu et al. 2019 (31) | All imaging examinations for HCC detection were performed within 2 months before starting the DAA treatment. | N/A |

**Supplementary Table 4.** Risk of bias assessment using the QUIPS tools (32)

| **Author (year)** | **1** | **2** | **3** | **4** | **5** | **6** | |
| --- | --- | --- | --- | --- | --- | --- | --- |
|  |  |  |  |  |  | **HR** | **OR** |
| Calvaruso et al. 2018 [2] |  |  |  |  |  |  |  |
| Ciancio et al. 2020 [3] |  |  |  |  |  |  |  |
| Conti F et al. 2016 [4] |  |  |  |  |  | N/A |  |
| Degasperi et al. 2019 [5] |  | N/A |  |  |  |  | N/A |
| Faillaci et al. 2018 [6] |  |  |  |  |  | N/A |  |
| Gardini et al. 2019 [7] |  | N/A |  |  |  |  | N/A |
| Ide et al. 2019 [8] |  |  |  |  |  |  |  |
| Janjua et al. 2020 [9] |  | N/A |  |  |  |  | N/A |
| Kanwal et al. 2020 [10] |  | N/A |  |  |  |  | N/A |
| Lleo et al. 2019 [11] |  |  |  |  |  |  |  |
| Lopez et al. 2020 [12] |  |  |  |  |  |  |  |
| Marino et al. 2019 [13] |  | N/A |  |  |  | N/A |  |
| Mecci et al. 2019 [14] |  |  |  |  |  |  |  |
| Mettke et al. 2017 [15] |  |  |  |  |  | N/A |  |
| Nagata et al. 2017 [16] |  |  |  |  |  |  | N/A |
| Nakagawa et al. 2020 [17] |  |  |  |  |  |  | N/A |
| Ogasawara et al. 2020 [18] |  | N/A |  |  |  |  | N/A |
| Ogawa et al. 2018 [19] |  |  |  |  |  |  | N/A |
| Ozeki et al. 2020 [20] |  | N/A |  |  |  |  |  |
| Pinero et al. 2019 [21] |  |  |  |  |  |  |  |
| Pons et al. 2020 [22] |  |  |  |  |  |  |  |
| Quaranta et al. 2020 [23] |  |  |  |  |  |  | N/A |
| Rinaldi et al. 2019 [24] |  |  |  |  |  |  |  |
| Romano et al. 2018 [25] |  |  |  |  |  |  |  |
| Sangiovanni et al. 2020 [26] |  |  |  |  |  |  | N/A |
| Shiha et al. 2020 [27] |  |  |  |  |  |  | N/A |
| Tani et al. 2020 [28] |  | N/A |  |  |  |  | N/A |
| Tayyab et al. 2020 [29] |  |  |  |  |  |  |  |
| Watanabe et al. 2019 [30] |  |  |  |  |  |  |  |
| Yoshimasu et al. 2019 [31] |  | N/A |  |  |  |  |  |

HR: hazard ratio; N/A: not attributable; OR: odds ratio

**Items in columns** 1: Study participation, 2: Study attrition, 3: Prognostic factor measurement, 4: Outcome measurement, 5: Study confounding, 6: Statistical analysis and reporting.

**Colors represent: Green**: low risk of bias, **Yellow**: moderate risk of bias, **Red:** high risk of bias

**Supplementary Table 5.** Parameters included in multivariate adjustment in each included article

| **Author (year)** | **Parameters included in multivariate adjustment** |
| --- | --- |
|  |  |
| Calvaruso et al. 2018 [2] | Not performed |
| Ciancio et al. 2020 [3] | Not performed |
| Conti F et al. 2016 [4] | Not performed |
| Degasperi et al. 2019 [5] | Gender, age, body mass index, hepatitis C genotype, alanine aminotransferase, bilirubin, international standardized ratio (INR), platelet count, MELD score, and esophageal varices, diabetes mellitus, g-glutamyl transferase, Child-Pugh-Turcotte score, a-fetoprotein, liver stiffness measurement, fibrosis-4 score, liver stiffness-spleen diameter-to-platelet ratio score |
| Faillaci et al. 2018 [6] | Not performed |
| Gardini et al. 2019 [7] | Not performed |
| Ide et al. 2019 [8] | Not performed |
| Janjua et al. 2020 [9] | Sustained virological response, previous interferon treatment, age, gender, hepatitis C genotype, material deprivation, cirrhosis, diabetes mellitus, injection drug use, problematic alcohol use, Elixhauser comorbidity index |
| Kanwal et al. 2020 [10] | Age, gender, race/ethnicity, cirrhosis, hepatitis C genotype, human immunodeficiency virus, diabetes, body mass index, alcohol use, other medical comorbidity, and healthcare use |
| Lleo et al. 2019 [11] | Not performed |
| Lopez et al. 2020 [12] | Not performed |
| Marino et al. 2019 [13] | Not performed |
| Mecci et al. 2019 [14] | Only significant – platelets, diabetes mellitus, nonmalignant lesions at baseline, albumin |
| Mettke et al. 2017 [15] | Not performed |
| Nagata et al. 2017 [16] | Not performed |
| Nakagawa et al. 2020 [17] | Age, gender, diabetes mellitus, hyperlipidemia, liver fibrosis, M2BPGi, a-fetoprotein, body mass index, obesity, fibrosis-4 score, aspartate aminotransferase to platelet ratio score, smoking, statin use |
| Ogasawara et al. 2020 [18] | Not performed |
| Ogawa et al. 2018 [19] | Not performed |
| Ozeki et al. 2020 [20] | Not performed |
| Pinero et al. 2019 [21] | Not performed |
| Pons et al. 2020 [22] | Not performed |
| Quaranta et al. 2020 [23] | Not performed |
| Rinaldi et al. 2019 [24] | Not performed |
| Romano et al. 2018 [25] | Gender, age, diabetes mellitus, obesity, cardiovascular disease, human immunodeficiency virus, hepatitis B surface antigen, hepatitis C genotype, fibroscan stiffness, aspartate aminotransferase to platelet ratio score, Child-Turcotte-Pugh score, type of direct-acting antiviral treatment, sustained virological response |
| Sangiovanni et al. 2020 [26] | Not performed |
| Shiha et al. 2020 [27] | Age, gender, diabetes mellitus, hypertension, overweight, a-fetoprotein, albumin, platelet count |
| Tani et al. 2020 [28] | Not performed |
| Tayyab et al. 2020 [29] | Age, gender, weight, hypertension, diabetes mellitus, hepatitis C genotype, sustained virologic response, type of direct-acting antiviral treatment |
| Watanabe et al. 2019 [30] | Not performed |
| Yoshimasu et al. 2019 [31] | Not performed |

# References

1. Page MJ, McKenzie JE, Bossuyt PM, Boutron I, Hoffmann TC, Mulrow CD, et al. The PRISMA 2020 statement: an updated guideline for reporting systematic reviews. *BMJ* (2021) 372:n71. doi: 10.1136/bmj.n71.

2. Calvaruso V, Cabibbo G, Cacciola I, Petta S, Madonia S, Bellia A, et al. Incidence of Hepatocellular Carcinoma in Patients With HCV-Associated Cirrhosis Treated With Direct-Acting Antiviral Agents. *Gastroenterology* (2018) 155(2):411-21. doi: 10.1053/j.gastro.2018.04.008. PubMed PMID: WOS:000440023400037.

3. Ciancio A, Giuseppe Ribaldone D, Dotta A, Giordanino C, Sacco M, Fagoonee S, et al. Long-term follow-up of diabetic and non-diabetic patients with chronic hepatitis c successfully treated with direct acting antiviral agents. *Liver Int* (2020) 41(2):276-87. Epub 2020/10/01. doi: 10.1111/liv.14676. PubMed PMID: 32998174.

4. Conti F, Buonfiglioli F, Scuteri A, Crespi C, Bolondi L, Caraceni P, et al. Early occurrence and recurrence of hepatocellular carcinoma in HCV-related cirrhosis treated with direct-acting antivirals. *J Hepatol* (2016) 65(4):727-33. doi: 10.1016/j.jhep.2016.06.015.

5. Degasperi E, D'Ambrosio R, Iavarone M, Sangiovanni A, Aghemo A, Soffredini R, et al. Factors Associated With Increased Risk of De Novo or Recurrent Hepatocellular Carcinoma in Patients With Cirrhosis Treated With Direct-Acting Antivirals for HCV Infection. *Clin Gastroenterol Hepatol* (2019) 17(6):1183-91. doi: 10.1016/j.cgh.2018.10.038.

6. Faillaci F, Marzi L, Critelli R, Milosa F, Schepis F, Turola E, et al. Liver Angiopoietin-2 Is a Key Predictor of De Novo or Recurrent Hepatocellular Cancer After Hepatitis C Virus Direct-Acting Antivirals. *Hepatology* (2018) 68(3):1010-24. doi: 10.1002/hep.29911.

7. Gardini AC, Foschi FG, Conti F, Petracci E, Marisi G, Buonfiglioli F, et al. Immune inflammation indicators and ALBI score to predict occurrence and recurrence of hepatocellular carcinoma in HCV-related cirrhosis treated with direct-acting antivirals. *Dig Liver Dis* (2018) 51(5):681–8. doi: 10.1016/j.dld.2018.01.055.

8. Ide T, Koga H, Nakano M, Hashimoto S, Yatsuhashi H, Higuchi N, et al. Direct-acting antiviral agents do not increase the incidence of hepatocellular carcinoma development: a prospective, multicenter study. *Hepatol Int* (2019) 13(3):293-301. Epub 2019/03/02. doi: 10.1007/s12072-019-09939-2. PubMed PMID: 30820753.

9. Janjua NZ, Wong SL, Darvishian M, Butt ZA, Yu AM, Binka M, et al. The impact of SVR from direct-acting antiviral- and interferon-based treatments for HCV on hepatocellular carcinoma risk. *J Viral Hepat* (2020) 27(8):781-93. doi: 10.1111/jvh.13295. PubMed PMID: WOS:000526175600001.

10. Kanwal F, Kramer JR, Asch SM, Cao Y, Li L, El-Serag HB. Long-Term Risk of Hepatocellular Carcinoma in HCV Patients Treated With Direct Acting Antiviral Agents. *Hepatology* (2020) 71(1):44-55. Epub 2019/06/22. doi: 10.1002/hep.30823. PubMed PMID: 31222774.

11. Lleo A, Aglitti A, Aghemo A, Maisonneuve P, Bruno S, Persico M, et al. Predictors of hepatocellular carcinoma in HCV cirrhotic patients treated with direct acting antivirals. *Dig Liver Dis* (2019) 51(2):310-7. doi: 10.1016/j.dld.2018.10.014.

12. Alonso López S, Manzano ML, Gea F, Gutiérrez ML, Ahumada AM, Devesa MJ, et al. A Model Based on Noninvasive Markers Predicts Very Low Hepatocellular Carcinoma Risk After Viral Response in Hepatitis C Virus–Advanced Fibrosis. *Hepatology* (2020) 72(6):1924-34. doi: 10.1002/hep.31588.

13. Mariño Z, Darnell A, Lens S, Sapena V, Díaz A, Belmonte E, et al. Time association between hepatitis C therapy and hepatocellular carcinoma emergence in cirrhosis: Relevance of non-characterized nodules. *J Hepatol* (2019) 70(5):874-84. doi: 10.1016/j.jhep.2019.01.005.

14. Mecci AJ, Kemos P, Leen C, Lawson A, Richardson P, Khakoo SI, et al. The association between hepatocellular carcinoma and direct-acting anti-viral treatment in patients with decompensated cirrhosis. *Aliment Pharmacol Ther* (2019) 50(2):204-14. doi: 10.1111/apt.15296.

15. Mettke F, Schlevogt B, Deterding K, Wranke A, Smith A, Port K, et al. Interferon-free therapy of chronic hepatitis C with direct-acting antivirals does not change the short-term risk for de novo hepatocellular carcinoma in patients with liver cirrhosis. *Aliment Pharmacol Ther* (2018) 47(4):516-25. Epub 2017/12/06. doi: 10.1111/apt.14427. PubMed PMID: 29205405.

16. Nagata H, Nakagawa M, Asahina Y, Sato A, Asano Y, Tsunoda T, et al. Effect of interferon-based and -free therapy on early occurrence and recurrence of hepatocellular carcinoma in chronic hepatitis C. *J Hepatol* (2017) 67(5):933-9. doi: 10.1016/j.jhep.2017.05.028. PubMed PMID: WOS:000413027500008.

17. Nakagawa M, Nawa N, Takeichi E, Shimizu T, Tsuchiya J, Sato A, et al. Mac-2 binding protein glycosylation isomer as a novel predictive biomarker for patient survival after hepatitis C virus eradication by DAAs. *J Gastroenterol* (2020) 55(10):990-9. Epub 2020/08/10. doi: 10.1007/s00535-020-01715-6. PubMed PMID: 32770465.

18. Ogasawara N, Saitoh S, Akuta N, Sezaki H, Suzuki F, Fujiyama S, et al. Advantage of liver stiffness measurement before and after direct-acting antiviral therapy to predict hepatocellular carcinoma and exacerbation of esophageal varices in chronic hepatitis C. *Hepatol Res* (2020) 50(4):426-38. Epub 2019/12/01. doi: 10.1111/hepr.13467. PubMed PMID: 31785120.

19. Ogawa E, Furusyo N, Nomura H, Dohmen K, Higashi N, Takahashi K, et al. Short-term risk of hepatocellular carcinoma after hepatitis C virus eradication following direct-acting anti-viral treatment. *Aliment Pharmacol Ther* (2018) 47(1):104-13. Epub 2017/10/17. doi: 10.1111/apt.14380. PubMed PMID: 29035002.

20. Ozeki I, Nakajima T, Suii H, Tatsumi R, Yamaguchi M, Arakawa T, et al. Predictors of hepatocellular carcinoma after hepatitis C virus eradication following direct-acting antiviral treatment: relationship with serum zinc. *J Clin Biochem Nutr* (2020) 66(3):245-52. Epub 2020/06/12. doi: 10.3164/jcbn.19-98. PubMed PMID: 32523252; PubMed Central PMCID: PMCPMC7263932.

21. Piñero F, Mendizabal M, Ridruejo E, Herz Wolff F, Ameigeiras B, Anders M, et al. Treatment with direct-acting antivirals for HCV decreases but does not eliminate the risk of hepatocellular carcinoma. *Liver Int* (2019) 39(6):1033-43. doi: 10.1111/liv.14041.

22. Pons M, Rodriguez-Tajes S, Esteban JI, Marino Z, Vargas V, Lens S, et al. Non-invasive prediction of liver-related events in patients with HCV-associated compensated advanced chronic liver disease after oral antivirals. *J Hepatol* (2020) 72(3):472-80. doi: 10.1016/j.jhep.2019.10.005. PubMed PMID: WOS:000514553200013.

23. Quaranta MG, Ferrigno L, Monti M, Filomia R, Biliotti E, Iannone A, et al. Advanced liver disease outcomes after hepatitis C eradication by human immunodeficiency virus infection in PITER cohort. *Hepatol Int* (2020) 14(3):362-72. doi: 10.1007/s12072-020-10034-0.

24. Rinaldi L, Perrella A, Guarino M, De Luca M, Piai G, Coppola N, et al. Incidence and risk factors of early HCC occurrence in HCV patients treated with direct acting antivirals: a prospective multicentre study. *J Transl Med* (2019) 17(1):292. Epub 2019/08/30. doi: 10.1186/s12967-019-2033-x. PubMed PMID: 31462268; PubMed Central PMCID: PMCPMC6712712.

25. Romano A, Angeli P, Piovesan S, Noventa F, Anastassopoulos G, Chemello L, et al. Newly diagnosed hepatocellular carcinoma in patients with advanced hepatitis C treated with DAAs: A prospective population study. *J Hepatol* (2018) 69(2):345-52. Epub 2018/03/20. doi: 10.1016/j.jhep.2018.03.009. PubMed PMID: 29551707.

26. Sangiovanni A, Alimenti E, Gattai R, Filomia R, Parente E, Valenti L, et al. Undefined/non-malignant hepatic nodules are associated with early occurrence of HCC in DAA-treated patients with HCV-related cirrhosis. *J Hepatol* (2020) 73(3):593-602. Epub 2020/04/04. doi: 10.1016/j.jhep.2020.03.030. PubMed PMID: 32243959.

27. Shiha G, Mousa N, Soliman R, Nnh Mikhail N, Adel Elbasiony M, Khattab M. Incidence of HCC in chronic hepatitis C patients with advanced hepatic fibrosis who achieved SVR following DAAs: A prospective study. *J Viral Hepat* (2020) 27(7):671-9. doi: 10.1111/jvh.13276.

28. Tani J, Morishita A, Sakamoto T, Takuma K, Nakahara M, Fujita K, et al. Simple scoring system for prediction of hepatocellular carcinoma occurrence after hepatitis C virus eradication by direct-acting antiviral treatment: All Kagawa Liver Disease Group Study. *Oncol Lett* (2020) 19(3):2205-12. Epub 2020/03/21. doi: 10.3892/ol.2020.11341. PubMed PMID: 32194718; PubMed Central PMCID: PMCPMC7038998.

29. Tayyab GUN, Rasool S, Nasir B, Rubi G, Abou-Samra AB, Butt AA. Hepatocellular carcinoma occurs frequently and early after treatment in HCV genotype 3 infected persons treated with DAA regimens. *BMC Gastroenterol* (2020) 20(1):93. doi: 10.1186/s12876-020-01249-4.

30. Watanabe T, Tokumoto Y, Joko K, Michitaka K, Horiike N, Tanaka Y, et al. Predictors of hepatocellular carcinoma occurrence after direct-acting antiviral therapy in patients with hepatitis C virus infection. *Hepatol Res* (2019) 49(2):136-46. Epub 2018/10/20. doi: 10.1111/hepr.13278. PubMed PMID: 30335208.

31. Yoshimasu Y, Furuichi Y, Kasai Y, Takeuchi H, Sugimoto K, Nakamura I, et al. Predictive factors for hepatocellular carcinoma occurrence or recurrence after direct-acting antiviral agents in patients with chronic hepatitis C. *J Gastrointestin Liver Dis* (2019) 28(1):63-71. Epub 2019/03/10. doi: 10.15403/jgld.2014.1121.281.hpc. PubMed PMID: 30851174.

32. Hayden JA, van der Windt DA, Cartwright JL, Côté P, Bombardier C. Assessing bias in studies of prognostic factors. *Ann Intern Med* (2013) 158(4):280-6. Epub 2013/02/20. doi: 10.7326/0003-4819-158-4-201302190-00009. PubMed PMID: 23420236.
